# Scientific Progress in Mapping the Relational Ecology of Early Child Development: A Systematic Scoping Review

**DOI:** 10.1007/s10567-025-00522-w

**Published:** 2025-04-25

**Authors:** Siobhan O’Dean, Elizabeth Spry, Tracy Evans-Whipp, Kayla Mansour, Rebecca Glauert, Craig A. Olsson, Tim Slade, the Australian Early Relational Health Network, Tim Slade, Jacqueline Allen, Cath Chamberlain, Juli Coffin, Donna Cross, Alex Fischer, Jacinta Francis, Matthew Fuller-Tyszkiewicz, Melissa Green, Ross Homel, Primrose Letcher, Jacqui A. Macdonald, Jennifer McIntosh, Shaun Mclaws, Siobhan M. O’Dean, Craig Olsson, Felicity Painter, Natasha Pearce, Naomi Priest, Lisa Ritland, Liz Spry, Sarah Whittle, Lu Zhang, Stephen R. Zubrick

**Affiliations:** 1 The Matilda Centre for Research in Mental Health and Substance Use, The University of Sydney, Sydney, New South Wales Australia; 2 Faculty of Health, SEED Lifespan Strategic Research Centre, School of Psychology, Deakin University, Melbourne, Victoria Australia; 3 University of Western Australia, Perth, Western Australia Australia; 4 Department of Paediatrics, Murdoch Children’s Research Institute, The University of Melbourne, Royal Children’s Hospital Campus, Melbourne, Victoria Australia

**Keywords:** Relational ecology, Early child development, Bioecological model of human development, Social network analysis, Scoping review

## Abstract

The development of secure relationships between children and their adult carers, across the earliest years of life, emerges within a multifaceted and complex relational ecology. Here we present findings from a systematic scoping review designed to map the extent to which the relational ecology of child-caregiver relationships across early life (from conception to age 3 years) has been studied. A first phase of the review searched for studies that used applied social network analysis (SNA) to measure the relational ecology. A second phase extended the scope to studies of associations between individual elements of the relational ecology and the early child-caregiver relationship. Searches were conducted between February and September, 2023, rerun in March 2025and in total, yielded 11,226 articles for screening. We found no studies using SNA to investigate the relational ecology of early child-caregiver relationship development. We did, however, find 122 studies that examined individual predictors across the relational ecosystem of the early child-caregiver relationship. Most studies focused on the family microsystem and in particular the mother–child relationship. Few studies examined other aspects of the microsystem, or higher levels of the relational ecosystem (meso-, exo- or macrosystems). Our findings highlight that much of the broader relational ecology of early child relational health development continues to be neglected in observational research. Future research should consider using novel methods like SNA to capture and explain interconnections between relationships at all levels of the relational ecology of early child-caregiver relationship development.

**Supplementary Information:**

The online version contains supplementary material available at 10.1007/s10567-025-00522-w.

## Introduction

**Fig. 1:**
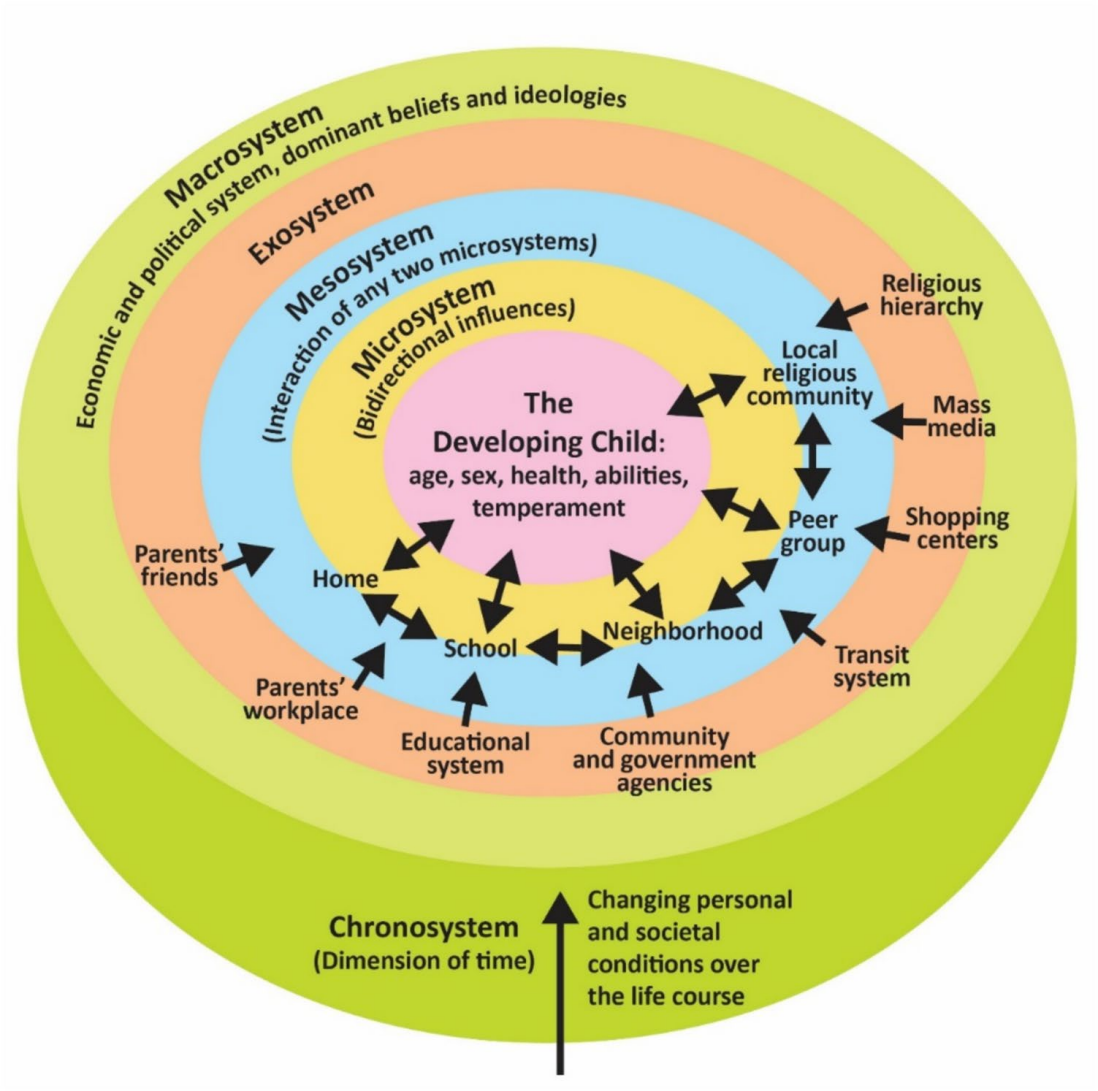
Social ecology of early child development (Image by Ian Joslin is licensed under CC BY 4.0)

Early child development emerges within a rich tapestry of relationships that sit within the various settings in which children live and grow, including (but not limited to) the family, early education and childcare, and neighbourhood and wider community settings, as well as the connections between them, and the institutions and societal values that support them (Bronfenbrenner, 1977, 2005). The qualities of relationships that support healthy development have been well described over the last century. Warm, empathic and responsive adult care creates the secure base (i.e., felt sense of safety) that children need in order to explore the world and to grow and develop in the process of exploration (Ainsworth et al., 1978). Child–adult carer interactions happen in multiple settings, the centre of which is the family microsystem, including immediate family (e.g., parents and siblings) as well as extended family (e.g., grandparents, aunts, uncles, cousins). Related microsystems include the early childcare microsystem (e.g., childcare workers and nannies), family, peer and community microsystems (e.g., family’s friends and neighbours), and broader government and non-government sponsored health systems (e.g., medical and allied health professional). Quality connections between microsystems (e.g., between parents and childcare) further strengthen the overall social ecology of early development forming another layer of the social ecology referred to as the mesosystem (see Fig. 1).

At broader levels sit wider social structures that indirectly influence proximal interactions occurring between children and adult carers. Examples include the quality of adult carer work environments, government financial supports and other social and healthcare services that form a protective exosystem. Broader still sit macrosystem factors that comprise the prevailing culture, attitudes and norms within which expectations about proximal interactions between children and their main adult carers are embedded and develop. At the broadest level sit chronosystem factors that encompass environmental changes that exert influence over the life course. These could include large-scale environmental events such as wars or pandemics.

In more recent years there has been an appreciation that the ecological influences on the developing child are much more complex than the simple nested configuration of social spheres shown in Fig. 1. For example, J. W. Neal and Neal (2013) propose that “the ecological environment is an overlapping arrangement of structures, each directly or indirectly connected to the others by the direct and indirect social interactions of their participants” (p.727). They further argue that these patterns of social interaction can be conceptualised as a social network and can thus be studied using methods developed within the field of social network analysis. Social network analysis is defined as an approach to data collection and analysis that captures information about the connections between entities (i.e. people, organisations, etc.). Egocentric networks (“ego-nets”) are most relevant as they describe the network of “alters” which form around a particular social “actor” (Crossley et al., 2015). This approach acknowledges that these actors are embedded within complex, interconnected social structures and that certain features of the structures themselves such as their density, reciprocity and centrality, can have a significant impact on outcomes. Our purpose in conducting this systematic scoping review was to map scientific progress in understanding the relational ecology of early development of the child-caregiver relationship; specifically, the extent to which studies have been conducted within systems, across systems, at all levels of the relational ecosystem. Given the remarkable similarities between bioecological concepts and concepts within social network analysis (SNA) (Z. P. Neal & Neal, 2017), we examined the use of SNA methods to model the relational ecology of early child development. A systematic scoping review approach was the most appropriate method of synthesis because (1) it accommodated variability in conceptualization of early relational health during early childhood, (2) the method is suitable for high-level mapping of longitudinal research on a broad range of potential influences including identification of research gaps, and (3) our aim was to describe the literature rather than report on the substantive findings of the identified studies (i.e. we do not quantify the nature and magnitude of the *association* between exposures and outcomes).

The aims of this systematic scoping review were threefold: (1) to determine the extent to which the methods and principles of SNA have been used to investigate the relational ecology of early child development, (2) to extend the scope of the review to studies that have examined any element of the early relational ecosystem using relationship specific measures, and (3) to summarise the types of relationships studied (e.g., mother, father, childcare worker, neighbour), the level of the bioecological model under investigation in these studies (i.e., micro-to-macrosystems), and methodological approaches used to study them (i.e., timing, mode of data collection and construct measured).

## Methods

The scoping review was guided by the Joanna Briggs Institute (JBI) approach to scoping reviews (Peters et al., 2020). Reporting utilised the Preferred Reporting Items for Systematic Reviews and Meta analyses Extension for Scoping Reviews (PRISMA-ScR; Tricco et al., 2018). The PRISMA-ScR Checklist is provided in the Supplementary Information (see Online Resource 1). This review was not pre-registered. As this review does not involve re-analysing data, ethics approval was not necessary.

The review was conducted in two phases. The first phase sought to identify studies that used applied SNA to measure the relational ecology of early childhood. The second phase was conducted in response to the outcome of the first phase (no identified studies) and extended the scope to studies examining longitudinal associations between any element of the early relational ecology (*exposure*) and the child-caregiver relationship (*outcome*). Both phases were restricted to studies of the relational ecology around the child–adult carer that were conducted within population-based longitudinal studies of child development. Both phases followed the JBI recommended PCC framework (Population, Concept, and Context; Pollock et al., 2023).

### Eligibility Criteria

*Participants*: The population of interest in this review are human infants from conception up to three years of age and their main adult carer or carers. Studies of general populations of infants, as well as those from generalised high-priority groups (such as low socioeconomic backgrounds**,** specific racial or ethnic groups, low-birth weight infants) were eligible for inclusion. Studies exclusively recruiting families from clinically indicated groups based on relational rupture (e.g. in families of infants in foster care) were excluded. Whilst in most cases the main caregivers were mothers or fathers, we included studies where other kin or non-kin adults played the primary caring role.

*Concept*: This scoping review considered longitudinal studies that examine any relational exposure associated with the quality of the child-caregiver relationships. We considered relational *exposures* from across the entire bioecology of the child–adult carer relationship:

- *Microsystem* relational exposures included measures of interactions or connections that happen within families (non-primary parent, grandparent/s, sibling/s, aunt/s, uncle/s, cousin/s, parent-parent interactions/relations, family functioning), early education and childcare (nannies/au-pair/childcare worker), peer (family friends, playmate) and community settings (neighbours).
- *Mesosystem* relational exposures included measures of interactions or connections that happen across microsystems (e.g., family educational setting, childcare-community, family community).
- *Exosystem* exposures included social systems that do not involve the child but that impact main carer capacities to provide care (enabling or disabling), including, but not limited to, main carer workplace experiences and other carer relevant social policies including government health and welfare supports.
- *Macrosystem* relational exposures included any effect of cultural master narratives (beliefs, values, and sources of prejudice and discrimination) particularly around sex/gender, ethnicity and socioeconomic position, that may indirectly impact the child-main-carer relationship.

Preconception relational exposures in parents’ life histories, including parents’ recall of attachment relationships in their own childhood, were excluded from this review because they were the focus of another review in this series (Macdonald et al., 2024).

The primary *outcome* of interest was the bond or relationship between infant and main carergiver/s including infant/child/foetal attachment, bonding and sensitivity. A condition for inclusion was that the measurement scale, or sub-scale, had an indication of the reciprocal relationship that could be either perceived (e.g., parental felt bond) or observed (e.g., attachment, or measures of contingent/responsive interaction that indicated the quality of the relationship dynamic). Indicators of child adjustment and behaviour were excluded, as were indicators of parenting styles and practices.

*Context*: This review considered empirical studies that have been conducted in any geographical location or setting.

### Types of Sources

Only original peer reviewed empirical articles were included. Systematic reviews, meta-analyses, dissertations, conference proceedings, commentaries, editorials, opinion papers and qualitative studies were excluded. Including studies with qualitative exposures and/or outcomes would have required a different methodological approach to account for more dynamic and complex qualitative data collection (e.g. focus group, observation and interview data centring on the participants’ understanding of relationships and how they impact infant development). We aimed to take a quantitative, data-driven, population health approach to this scoping review. As such, qualitative research was outside of the scope of the current review.

### Search Strategy

The search strategies were developed in consultation with a research librarian.

*Phase 1 search strategy*: The first phase of the review examined the use of applied SNA to study the early relational ecology. Search terms combined three concepts: (1) child and family relational ecology; (2) child age in the period from conception up to three years of age (up to 48 months postpartum); and (3) use of SNA study methodology captured by the terms “social network”, "ego network”, “sociogram”, “graph theory”, “structural network” (based on search terms described in Collonnaz et al. (2022). Electronic databases (MEDLINE [EBSCOhost], PsycINFO [EBSCOhost], Embase [EBSCOhost] were searched for relevant evidence. The search strategies were adapted for each included database and information source and restricted to English.

*Phase 2 Search Strategy*: The second phase did not apply an SNA restriction. Search terms combined three concepts: (1) child and family relational ecology; (2) infant age in the period from conception up to three years of age (up to 48 months postpartum); and (3) population-based longitudinal study design. The full electronic search strategy is provided in Supplementary Information (see Online Resource 2). Electronic databases (MEDLINE [EBSCOhost], PsycINFO [EBSCOhost], Embase [EBSCOhost] were searched for relevant evidence. The search strategy was adapted for each included database and information source and restricted to English.

The phase 1 search was conducted in February 2023 and the phase 2 search in September, 2023. Updates to both phase 1 and phase 2 searches were conducted in March 2025. Both searches were limited to human populations and English language and peer reviewed publications. No date limitations were applied.

### Study Selection

**Fig. 2:**
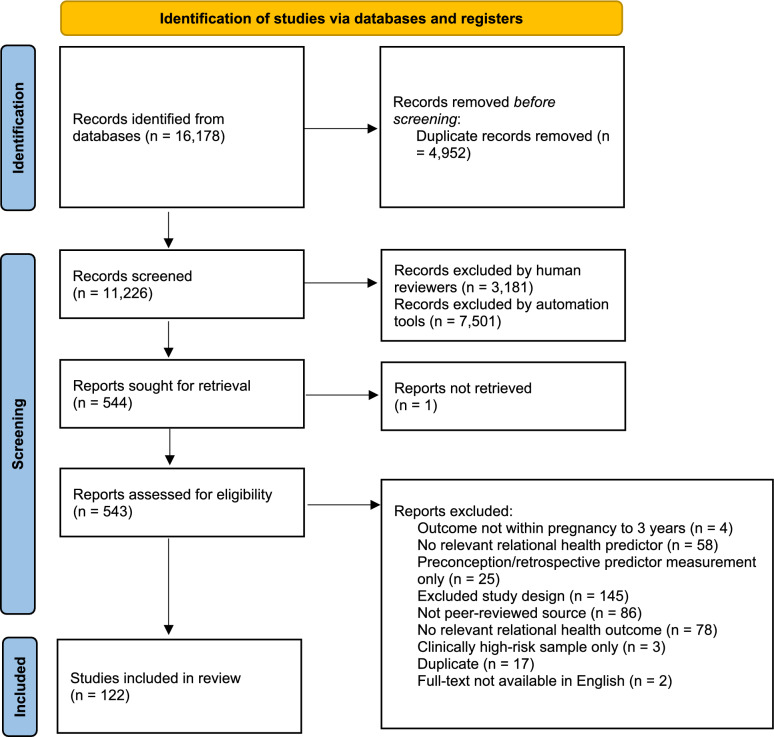
PRISMA Flow diagram of the study selection process for the phase 2 search strategy

The smaller number of titles and abstracts found in the first phase of the review (see results) were manually screened for relevance by two independent reviewers. The larger number of titles and abstracts identified in the second phase of the review (see results) were screened using an AI facilitated screening tool LitQuest (Grbin et al., 2022). All duplicates were removed. Double screening against eligibility criteria was conducted by a team of five independent reviewers until a stop-point was indicated by LitQuest (Fuller-Tyszkiewicz et al., 2024). Screening disagreements were discussed amongst the research team to reach a consensus decision. The full text of potentially relevant articles was then assessed by two independent reviewers to ensure the article met inclusion criteria. After double screening 68% of articles, the inter-rater reliability was assessed on a sample of 20 unscreened articles and found to be 95% and the remaining articles were single screened. The reasons for exclusion at full text review are identified in the PRISMA flowchart of articles screened for inclusion in the scoping review (Fig. 2).

### Data Extraction

No studies identified in phase 1 of the review met criteria for inclusion. Consequently, no data extraction was needed. For phase 2, data from identified studies were tabulated into a standardized data extraction form detailing: (1) study and sample characteristics, and (2) exposure and outcome variables, measures and assessment mode/timing. Data were only extracted for variables relevant to the review aims. The data extraction form was pilot tested on five included studies to assess consistency of the extraction as well as appropriateness and usability of the form. Following pilot testing, data were extracted from included studies by one of four reviewers, with 10% cross-checked by another.

### Data Collation and Analysis

Studies were classified according to year of publication, location (World Bank income classification and World Bank region, (*World Bank Income Categories*, n.d.), sample size, and whether they were designed as general population samples. Relational *exposures* were classified by timing (i.e., child’s age or mother’s pregnancy trimester), mode (e.g., parental report, researcher observed), construct (e.g., caregiver perceived bond, social support) and level of the bioecological system. Relational *outcome* measures of the quality of the child-caregiver relationship were classified according to timing and mode of assessment, carer reported on, and type of interaction (child behavioural initiatives [e.g., child attachment], parent behavioural initiatives [e.g., main carer attachment or bonding, responsive caregiving or carer-child interaction quality]). The extracted data and analysis code are available at [OSF link TBC]. Descriptive analyses of the extracted data were undertaken using Stata/SE 18.0 (StataCorp, 2023).

### Establishing the Living Review

Our intention is to monitor the emergence of new literature in this field following Cochrane and other guidance on living reviews (Elliott et al., 2021; Iannizzi et al., 2023; Synnot, 2019). We plan to conduct periodic updates of this review as a living review. For this, searches will be rerun and output uploaded into LitQuest to allow newly retrieved articles to be screened against the established eligibility criteria. Data will be extracted from included articles using the same methodology described here and synthesised with previous evidence. Timing of publication updates will be based on the impact of the new data on the review conclusions. At each update, we will assess the feasibility and need for continuation of the living mode.

## Results

The first phase of the review identified 67 population-based cohort studies that applied the principles and methods of social network analysis to study the relational ecology of child development. On double screening, none of these studies examined associations between the relational ecology and the quality of the early child–adult carer relationship between conception and three years of age. The extended second phase of the search returned 11,226 articles after removal of duplicates. At title and abstract screening in LitQuest, 3725 articles (33.2%) were manually screened, with 3181 excluded. The remaining 7501 articles were excluded by LitQuest. Full text screening was carried out on 543 articles and 122 articles were deemed eligible for inclusion. Screening and eligibility are reflected in the PRISMA flowchart (Fig. 2). Study details of the 122 included articles are provided in Online Resource 3.

### Study Characteristics

Table 1 summarises the frequency and proportion of included study characteristics (year of publication, region, population and sample size). Identified articles were published between 1981 and 2025, with half published in the last 10 years. Of the 122 studies included, around half reported on samples from North America, around one in three were from Europe & Central Asia, and around one in ten reported on samples from East Asia/Pacific. Only ten per cent of studies in total were conducted across remaining regions of Latin America/Carribean (*k* = 5), Middle East/North Africa (*k* = 4), South Asia (*k* = 2) and Sub-Saharan Africa (*k* = 2) combined. Most (87.7%) studies used samples from high income countries. The remaining studies were from middle income countries. No studies used data from low-income countries.

**Table 1:** Frequency and proportion of study characteristics (*k* = 122)

Characteristic		Number of studies	Proportion (of total *k*)
Sample size	< 100	57	46.7%
	100–499	47	38.5%
	500–999	13	10.7%
	1000–4999	4	3.3%
	> = 5000	1	0.8%
Publication year	1981–1990	6	4.9%
	1991–2000	15	12.3%
	2001–2005	9	7.4%
	2006–2010	8	6.6%
	2011–2015	23	18.9%
	2016–2020	25	20.5%
	2021–2025	36	29.5%
Population	Subgroup	40	32.8%
	General population	82	67.2%
World Bank income	LIC	0	0.0%
	MIC	15	12.3%
	HIC	107	87.7%
World Bank region	East Asia and Pacific	12	9.8%
	Europe and Central Asia	38	31.1%
	Latin America and Caribbean	5	4.1%
	Middle East and North Africa	4	3.3%
	North America	59	48.4%
	South Asia	2	1.6%
	Sub-Saharan Africa	2	1.6%

Two thirds of included studies were designed as general population samples. The remaining studies investigated subgroup population samples (e.g., first-time parents, adolescent parents, socio-demographically disadvantaged, pre-term birth/NICU). Just under half of all studies had sample sizes of less than 100, around one in three studies had sample sizes between 100 and 499, and around 15 percent had sample sizes over 500.

### Outcomes

Table 2 summarises the frequency and proportion of outcome constructs and measurement characteristics in included studies. The child-caregiver relationship was assessed across four separate constructs that were derived from the literature:

**Table 2:** Frequency and proportion of outcome constructs and measurement characteristics (*k* = 122)

Characteristic		Number of studies	Proportion (of total *k*)*
Construct	Child attachment security	33	27.0%
	Caregiver felt bond	46	37.7%
	Responsive caregiving	38	31.1%
	Caregiver-child interaction	24	19.7%
Caregiver	Mother	106	86.9%
	Father	31	25.4%
	Both/mixed	9	7.4%
	Teacher	1	0.8%
Timing of assessment	Antenatal	7	5.7%
	Postnatal	115	94.3%
Trimester (if antenatal)	2	1	0.8%
	3	4	3.3%
Child age (if postnatal)	< 1 month	6	4.9%
	> = 1, < 6 months	42	34.4%
	> = 6, < 12 months	26	21.3%
	> = 12, < 24 months	51	41.8%
	> = 24, < 48 months	17	13.9%
Measurement mode	Maternal report	42	34.4%
	Paternal report	11	9.0%
	Researcher observation	79	64.8%

^*^The proportion of the total number of studies with each level of a characteristic often do not add up to 100%; this is because of missing data and/or studies that included more than one level of a characteristic (for example, studies that assessed an outcome at multiple timepoints, or studies that assessed more than one outcome construct)

#### Child Attachment Security

This construct focussed on the child’s attachment-related behaviour towards the caregiver. Thirty-three studies (27.0%) reported on the impact of relational exposures on infant attachment to their caregiver. Child attachment was most commonly measured through researcher observation using procedures like the Ainsworth Strange Situation task (Ainsworth et al., 1978). From these interactions, observers coded the behaviours of the child in response to social interactions with their caregiver and a stranger. Then, from the observations in these situations, researchers assign a type of attachment style of the children (i.e., Secure, Anxious, Avoidant, Disorganised).

#### Caregiver Felt Bond

 This construct focussed on the caregiver’s perceptions of their own felt bond to the child. Forty-six studies (37.7%) assessed the felt bond between carers and children. This construct was primarily measured through self-report questionnaires such as the Maternal Attachment Inventory (Müller, 1994) and Pre and Postnatal Bonding Scale (Cuijlits et al., 2016), or Maternal and Paternal Postnatal Attachment Scale (Condon & Corkindale, 1998).

#### Responsive Caregiving

 This construct focussed on the caregiver’s behaviour towards the child, and in particular on sensitive, contingent, and responsive care. Thirty-eight studies (31.1%) assessed the effects of relational exposures on responsive caregiving. This construct was primarily measured using researcher observation of interactions between caregiver and child. Interactions were coded by observers for qualities like sensitivity, responsivity, and emotional availability using paradigms that usually involve a recorded play period which is then micro-behaviourally coded using a standardised coding system such as provided for the Emotional Availability Scales (Biringen et al., 2000) or the Parent Caregiver Involvement Scale (Farran et al., 1986). However, there were exceptions to this. For example, one study used a self-report measure of emotional availability, the 36 item Emotional Availability – Self Report (Vliegen et al., 2009) which asked questions specifically pertaining to the dyad of parent–child (Salo et al., 2021).

#### Child-caregiver Interaction

 This last construct focussed on the quality of dynamic interactions between the child-caregiver dyad or triad. Twenty-four (19.7%) studies investigated the effects of relational exposures on the quality of interactions between caregiver and child. As above, these outcomes were derived from researcher observation and coding of interactions between the caregiver and child.

#### Outcome Caregiver

 The bond between the child and the mother was the most reported relationship (*k* = 106). Thirty-one studies examining father-child bond. Nine studies examined both parents, and 1 assessed the teacher–child bond. No other child-primary caregiver relationships were examined in included studies.

#### Timing of Outcome Assessment

 Few (5.7%) measured child-caregiver relational outcomes antenatally. In contrast, one-hundred-and-fifteen studies measured outcomes postnatally (94.3%). Over half of these studies measured relational outcomes within the first year of the infant’s life and a further 51 studies measured outcomes between 12 and 24 months. Few studies investigated the impacts of relational exposures on studies beyond 24 months of age (*k* = 17).

#### Outcome Measurement Mode

 Overall, the majority (around two thirds) of studies included an observational assessment of the outcome. Around one in three studies included maternally reported outcomes, and only one in ten included paternally reported outcomes.

### Exposures

Table 3 summarises the frequency and proportion of predictor constructs, ecological levels, and measurement characteristics in included studies.

**Table 3:** Frequency and proportion of exposure ecological levels, constructs, and measurement characteristics (*k* = 122)

Characteristic		Number of studies	Proportion (of total *k*)*
Ecological level	Microsystem	104	85.2%
	Mesosystem	17	13.9%
	Exosystem	5	4.1%
	Macrosystem	5	4.1%
Construct	Microsystem		
	Child attachment security	10	8.2%
	Caregiver felt bond	37	30.3%
	Responsive caregiving	38	31.1%
	Caregiver-child interaction	10	8.2%
	Co-caregiving	5	4.1%
	Caregiver-partner relationship	25	20.5%
	Intimate partner violence	3	2.5%
	Caregiver-sibling relationship	2	1.6%
	Mesosystem		
	Social stress	3	2.5%
	Social support	15	12.3%
	Exosystem		
	Perceived neighbourhood safety	1	0.8%
	Early discharge from hospital	1	0.8%
	Rooming in	2	1.6%
	Job satisfaction	1	0.8%
	Macrosystem		
	Cultural differences	5	4.1%
Timing of assessment	Antenatal	54	44.3%
	Postnatal	74	60.7%
Trimester (if antenatal)	1	4	3.3%
	2	16	13.1%
	3	25	20.5%
Child age (if postnatal)	< 1 month	8	6.6%
	> = 1, < 6 months	34	27.9%
	> = 6, < 12 months	25	20.5%
	> = 12, < 24 months	17	13.9%
	> = 24, < 48 months	4	3.3%
Measurement mode	Maternal report	61	50.0%
	Paternal report	17	13.9%
	Parent report (unspecified/mixed)	1	0.8%
	Researcher observation	53	43.4%
	Other	10	8.2%

^*^The proportion of the total number of studies with each level of a characteristic often do not add up to 100%; this is because of missing data and/or studies that included more than one level of a characteristic (for example, studies that assessed an outcome at multiple timepoints, or studies that assessed more than one outcome construct)

*Ecological Level*: The majority of studies (*k* = 104; 85.2%) reported on relational exposures within the family *microsystem*. Of the studies that examined microsystem relational exposures, the most commonly measured exposures were the caregiver felt bond to their child (*k* = 37), responsive caregiving (*k* = 38), and caregiver-partner relationship (e.g., relationship/marital satisfaction or quality, or intimate partner violence) (*k* = 28). Ten studies measured child attachment security, through procedures like the Ainsworth Strange Situation paradigm, and 10 studies measured child-caregiver interaction using researcher observed interaction. Less commonly measured were caregiver-sibling relationship (*k* = 2) and co-caregiving (*k* = 5).

Seventeen studies investigated the effects of *mesosystem* relational processes on the quality of the child-main-carer relationship from conception to three. Mesosystem relational exposures included those that measured social support and social stress. Social support was often measured as the amount of perceived support received from extended family members and/or friends. This was often measured using self-report items derived specifically for the study, however some studies used validated scales such as the Norbeck Social Support Questionnaire (NSQ; Norbeck, 1984) and the Multidimensional Scale of Perceived Social Support (MSPSS; Wilcox, 2010). Social stress was measured in three studies using questionnaires like Brief Risk and Resilience Battery (Moore et al., 2020), and the familial and social strain subscale of the Impact on Family Scale (Stein & Riessman, 1980).

Five studies looked at *exosystem* relational exposures. These included studies that investigated the effects of hospital level policies on the development of mother–child bond. One of these studies looked at early discharge from hospital on mother child interaction quality (Britton et al., 1999) and the others investigated the effects of ‘rooming in’ procedures on postpartum maternal bonding to child (Handelzalts et al., 2021). A further study investigated the effects of carer job satisfaction on later child-caregiver bond, and one study looked at the effects of perceived neighbourhood safety on the child-caregiver bond.

Five studies investigated *macrosystem* relational exposures of child-main-carer bond. These studies assessed cultural differences and their association with child-caregiver relationship through cross-country comparison (e.g., comparing families from Cameroon to Germany, Lamm et al., 2015) or through comparison of indigenous and non-indigenous groups within a country (Taverna et al., 2024). No studies examined repeated relational exposures over time within chronosystem.

*Timing of Exposure Assessment:* Just under half of the included studies assessed the exposure antenatally. Of those that reported on the timing of the assessment within the pregnancy, third trimester assessments were the most frequent, followed by second trimester. Few studies included assessments in trimester 1. Nearly two thirds of the included studies assessed the exposure postnatally. The largest proportions of postnatal assessments occurred within the first year of life, followed by those conducted whilst the child was one year of age. Only 4 included studies assessed predictors whilst children were 2–3 years of age.

*Mode of Predictor Assessment:* Most relational exposures were measured through maternal self-report (k = 61, 50.0%) or researcher observation (*k* = 53, 43.4%), with 17 studies measured through paternal report (13.9%), one unspecified parental report, and 10 other methods (e.g. geographical location of recruitment; Lamm et al., 2015).

## Discussion

In this scoping review we aimed to map scientific progress in understanding the relational ecology of early child development, from families to community settings, connections between them, and structures that support them. We found that most studies focused on the family microsystem, with a particular focus on the mother–child relationship, and an occasional focus on the father-child relationship. Relatively few studies focused on other aspects of the microsystem, or higher levels of the relational ecosystem (meso-, exo- or macrosystems). Overall, it is evident that the complete relational ecosystem is poorly studied and that most studies are concentrated within the family microsystem. Moreover, the nature of the way these relationships were measured had substantial heterogeneity. The neglect of the broader relational ecology of early child development may put practitioner and social policy makers at risk of placing too much responsibility on adult carers themselves, within family settings in particular, and not enough on the wider social structures that support adult carers to care for children. These gaps in our collective knowledge of the early relational ecosystem requires urgent attention in future observational research.

The dearth of research in these areas may be because of the inherent challenges in measuring and analysing more diffuse and complex social environments. Indeed, the complexity and dynamism of these interactions necessitates innovative research designs and methodologies that are able to account for the interrelated nature of the social systems effecting early relational health and how these associations develop and change over the life course. As briefly outlined in the introduction, one possible way to capture these associations is using social network data collection and analysis methods.

Our initial searches did not identify any empirical research using social network methodologies to study relational health outcomes. However, we did encounter a conceptual approach to integrating social network analysis within a bioecological framework (e.g., Neal & Neal, 2017). Recognising that the ecological influences impacting an individual (or, in the current case, an infant-adult carer dyad) can be more complex than a simple nested configuration of social systems, this conceptual approach proposes that ecological influences are networked in complex and intersecting ways. The complexity of these networked ecological systems can be studied using well formulated social network data collection and analysis methodologies (Crossley et al., 2015; Kadushin, 2012). These models offer promising avenues for future research, particularly in understanding how different social systems interact to influence the infant-adult carer bond.

Additionally, future research could focus on examining how the microsystem of the family interacts with broader macrosystem influences, such as cultural norms and societal values. For instance, comparative studies exploring family dynamics within different cultural contexts (e.g., Italian versus Jewish cultures within the same geographical location) could provide insights into how macrosystem influences like cultural values shape child-caregiver relationships and the broader social ecology. Ensuring that future studies use population-representative samples will be critical for enabling generalization of findings across diverse populations. This will also ensure that interventions designed based on findings from such studies can effectively address the needs of different demographic and cultural groups, particularly those that might currently be underrepresented in the literature.

Bronfenbrenner’s model conceptualises the microsystem as the immediate environment in which the child directly interacts. Traditionally, this includes aspects of the family, school and peer environments. However, there has been increasing integration of technology in daily life through digital spaces (e.g., social media, virtual communication platforms and online education). These digital environments are becoming commonplace as part of the child and their caregiver’s immediate environment and may act as another aspect of the microsystem that influences a child’s social and emotional development (Navarro & Tudge, 2023). For instance, the nature and quality of child-caregiver interactions within digital platforms, the ways in which carers use technology in the home, and the cultural attitudes toward digital device use could each shape child development. As such, the introduction of a digital microsystem raises new questions about how the use of technology can strengthen or weaken relational bonds and health between children and their adult carers.

## Limitations

Our review has some limitations worth noting. First, this review focussed on general population samples and excluded clinically indicated samples (e.g., studies with sampling based on domestic violence or foster care), which may provide important insights into how extreme relational disruptions affect the child-caregiver relationship. By focusing only on general populations, the review may overlook critical dynamics present in high-risk groups, limiting the broader applicability of the findings. Moreover, the exclusion of qualitative studies may limit nuance in our understanding of the dynamics of child-caregiver relationships, that may be difficult to capture through quantitative methods alone. Similarly, many of the exposures identified in this review were based on subjective measurement, as opposed to objective contextual measures of exposures. However, it is important to note that perceptions of ecological factors may indeed be a more powerful influence on child-caregiver relational health than objective factors (Bronfenbrenner, 2005). Finally, our review included only published literature, which may introduce publication bias, skewing our understanding of the full scope of research in this area.

## Actionable insights

- Recommendation 1: The current research on early relational health has had a myopic focus on the influence of immediate family microsystem, with very little research focusing on the influence of other systems and structures. To better understand the child development over time, it is critical to broaden this focus to capture these dynamic interactions between the wider social ecology and the child–adult carer dyad. Given that these systems are in a constant state of change and evolution, we recommend increasing investment in longitudinal research that accurately captures the evolving nature of relationships within the social ecology. This research should prioritise tracking the development of, and changes in, relational health and social networks across developmental stages from early childhood into adulthood to provide a more comprehensive picture of how these concepts and contexts shift over time.
- Recommendation 2: We recommend the use of more modern methods to study these broader systems. Specifically, conducting a social network research study to characterise the networked social ‘settings’ that work independently and synergistically to influence child-caregiver relationships. By viewing relational health from a social network lens and thinking differently about how the major social influences of the parent–child relationship are structured and arranged, the power to intervene will be increased.
- Recommendation 3: Develop and implement measures that capture both the health of the social network at all levels of the social ecology and the quality of the child-caregiver relationship, that are dynamic and developmentally appropriate. These measures should be practical to implement, so they can be integrated into nationally representative studies to provide a more comprehensive understanding of relational health dynamics.
- Recommendation 4: Digital environments, or virtual microsystems (Navarro & Tudge, 2023), need to be understood in their complexity. We should aim to understand how children and their carers are interacting with digital technologies now, as well being able to capture how it changes dynamically and rapidly over time. We recommend taking a broader perspective on the digital world that not only measures the potentially detrimental effects, but also explores how the digital social realm can contribute to wellbeing and flourishing of relationships and relational health.

## Supplementary Information

Below is the link to the electronic supplementary material.

Supplementary file1 (DOCX 107 KB)

Supplementary file2 (DOCX 25 KB)

Supplementary file3 (DOCX 592 KB)

## Data Availability

No datasets were generated or analysed during the current study.
