# Supplementary material for: Scientific Progress in Mapping the Relational Ecology of Early Child Development: A Systematic Scoping Review
Source: Clin Child Fam Psychol Rev. 2025 Apr 25;29(2):200–12. doi: 10.1007/s10567-025-00522-w (PMC13282221; doi:10.1007/s10567-025-00522-w)
Supplement: Supplementary file 3 — Supplementary file3 (DOCX 592 KB) [file 10567_2025_522_MOESM3_ESM.docx]

**Online Resource 3**

Characteristics and reference list of each included study (*k* = 122)

| **Study** |  | **Outcome** |  |  |  | **Predictor** |  |  |  |
| --- | --- | --- | --- | --- | --- | --- | --- | --- | --- |
| Author, year | Region, sample N | Construct | Caregiver | Timing | Mode | Ecological level | Construct | Timing | Mode |
| Abbasoglu et al. (2022) | Europe & Central Asia, N=600 | Caregiver felt bond | Mother | 1-6 months postpartum | Maternal report | Microsystem | Caregiver felt bond | Trimester 3 | Maternal report |
| Ahlfs-Dunn et al. (2022) | North America, N=120 | Child attachment | Mother | 12-24 months postpartum | Researcher observation | Microsystem | Caregiver felt bond, intimate partner violence | Trimester 3 | Maternal report |
| Altenburger et al. (2018) | North America, N=182 | Responsive caregiving | Father | 6-12 months postpartum | Researcher observation | Microsystem | Co-caregiving, responsive caregiving | 1-6 months postpartum | Paternal report, researcher observation |
| Baldisserotto & Filha (2023) | Latin America & Caribbean, N=415 | Caregiver felt bond | Mother | Trimester 3 | Maternal report | Mesosystem | Social support | Trimesters 1 and 2 | Maternal report |
| Bang et al. (2020) | East Asia & Pacific, N=97 | Caregiver felt bond | Mother | Trimester 3, trimesters 2 and 3 | Maternal report | Microsystem | Caregiver felt bond | Trimester 2, trimesters 2 and 3 | Maternal report |
| Barnett et al. (2006) | North America, N=50 | Child attachment | Mother | 24-48 months postpartum | Researcher observation | Mesosystem, microsystem | Child attachment, social stress | 24-48 months postpartum | Maternal report, researcher observation |
| Belsky et al. (1984) | North America, N=72 | Responsive caregiving | Father, mother | 1-6 months postpartum, 6-12 months postpartum | Researcher observation | Microsystem | Responsive caregiving | 1-6 months postpartum | Researcher observation |
| Belsky & Fearon (2002) | North America, N=1053 | Responsive caregiving | Mother | 24-48 months postpartum | Researcher observation | Microsystem | Child attachment | 12-24 months postpartum | Researcher observation |
| Bendel-Stenzel et al. (2023) | North America, N=306 | Caregiver-child interaction | Father, mother | 24-48 months postpartum | Researcher observation | Microsystem | Child attachment | 12-24 months postpartum | Researcher observation |
| Bernier et al. (2014) | North America, N=63 | Caregiver-child interaction | Father | 12-24 months postpartum | Researcher observation | Microsystem | Caregiver-partner relationship | 12-24 months postpartum | Paternal report |
| Bilgin & Wolke (2022) | Europe & Central Asia, N=73 | Child attachment, responsive caregiving | Mother | 12-24 months postpartum | Maternal report, researcher observation | Microsystem | Caregiver felt bond, responsive caregiving | 1-6 months postpartum | Maternal report, researcher observation |
| Bloom (1995) | North America, N=49 | Caregiver felt bond | Mother | Trimesters 1, 2 and 3 | Maternal report | Microsystem | Caregiver felt bond | Trimesters 1, 2 and 3 | Maternal report |
| Boekhorst et al. (2024) | Europe & Central Asia, N=1003 | Caregiver felt bond | Mother | 12-24 months postpartum, 6-12 months postpartum | Maternal report | Microsystem | Caregiver felt bond | Trimester 3, 1-6 months postpartum, 6-12 months postpartum | Maternal report |
| Bornstein et al. (2012) | North America, N=58 | Responsive caregiving | Mother | 12-24 months postpartum | Researcher observation | Mesosystem, microsystem | Caregiver-partner relationship, responsive caregiving, social support | 1-6 months postpartum | Maternal report, researcher observation |
| Bornstein et al. (2016) | North America, N=61 | Responsive caregiving | Mother | 1-6 months postpartum | Researcher observation | Microsystem | Caregiver-sibling relationship | 1-6 months postpartum | Researcher observation |
| Bornstein et al. (2019) | North America, N=55 | Responsive caregiving | Mother | 12-24 months postpartum | Researcher observation | Microsystem | Caregiver-sibling relationship | 12-24 months postpartum | Researcher observation |
| Branjerdporn et al. (2020) | East Asia & Pacific, N=60 | Caregiver felt bond | Mother | 1-6 months postpartum | Maternal report | Microsystem | Caregiver felt bond | Antenatal (unspecified) | Maternal report |
| Braungart-Rieker et al. (2001) | North America, N=94 | Child attachment | Mother | 12-24 months postpartum | Researcher observation | Microsystem | Responsive caregiving | 1-6 months postpartum | Researcher observation |
| Britton et al. (1999) | North America, N=146 | Caregiver-child interaction, child attachment | Mother | 1-6 months postpartum, 12-24 months postpartum, 6-12 months postpartum | Researcher observation | Exosystem | Early discharge from hospital | <1 month postpartum | Other |
| Capuzzi (1989) | North America, N=20 | Caregiver felt bond | Mother | 1-6 months postpartum | Researcher observation | Mesosystem | Social support | Antenatal (unspecified) | Maternal report |
| Cárcamo et al. (2016) | Latin America & Caribbean, N=95 | Caregiver felt bond, responsive caregiving | Mother | 6-12 months postpartum | Researcher observation | Microsystem | Child attachment, responsive caregiving | 6-12 months postpartum | Researcher observation |
| Cataudella et al. (2022) | Europe & Central Asia, N=123 | Caregiver felt bond | Mother | 1-6 months postpartum | Maternal report | Mesosystem, microsystem | Caregiver felt bond, caregiver-partner relationship, social support | Trimesters 2 and 3 | Maternal report |
| Célia et al. (2018) | North America, N=56 | Responsive caregiving | Mother | 12-24 months postpartum | Researcher observation | Microsystem | Responsive caregiving | 1-6 months postpartum, 12-24 months postpartum | Researcher observation |
| Chibucos & Kail (1981) | North America, N=19 | Child attachment, responsive caregiving | Father | 6-12 months postpartum | Researcher observation | Microsystem | Caregiver-child interaction, responsive caregiving | 1-6 months postpartum | Researcher observation |
| Chtabou et al. (2024) | Middle East & North Africa, N=234 | Caregiver felt bond | Mother | 12-24 months postpartum | Maternal report | Microsystem | Caregiver felt bond | <1 month postpartum | Maternal report |
| Çınar et al. (2022) | Europe & Central Asia, N=150 | Caregiver felt bond | Mother | 1-6 months postpartum | Maternal report | Microsystem | Caregiver felt bond | Antenatal (unspecified) | Maternal report |
| Condon et al. (2013) | North America, N=235 | Caregiver felt bond | Father | 12-24 months postpartum, 6-12 months postpartum | Paternal report | Microsystem | Caregiver felt bond, caregiver-partner relationship | Trimester 2, 6-12 months postpartum | Paternal report |
| Cox (1992) | North America, N=36 | Child attachment | Father, mother | 12-24 months postpartum | Researcher observation | Microsystem | Caregiver-child interaction | 1-6 months postpartum | Researcher observation |
| Crockenberg & Leerkes (2003) | North America, N=92 | Responsive caregiving | Mother | 1-6 months postpartum | Researcher observation | Microsystem | Caregiver-partner relationship | Trimester 3 | Maternal report |
| Cuijlits et al. (2019) | Europe & Central Asia, N=793 | Caregiver felt bond | Mother | 6-12 months postpartum | Maternal report | Microsystem | Caregiver felt bond, caregiver-partner relationship | Trimester 3 | Maternal report |
| da Rosa et al. (2021) | Latin America & Caribbean, N=839 | Caregiver felt bond | Mother | Antenatal (unspecified) | Maternal report | Mesosystem, microsystem | Caregiver-partner relationship, social support | Antenatal (unspecified) | Maternal report |
| Damato (2004) | North America, N=142 | Caregiver felt bond | Mother | 1-6 months postpartum | Maternal report | Microsystem | Caregiver felt bond | Antenatal (unspecified) | Maternal report |
| Dayton et al. (2010) | North America, N=164 | Responsive caregiving | Mother | 12-24 months postpartum | Researcher observation | Microsystem | Caregiver felt bond | Trimester 3 | Maternal report |
| Diniz et al. (2016) | Latin America & Caribbean, N=39 | Responsive caregiving | Mother | 6-12 months postpartum | Researcher observation | Mesosystem, microsystem | Responsive caregiving, social support | 1-6 months postpartum | Maternal report |
| Doyle et al. (2023) | East Asia & Pacific, N=357 | Caregiver felt bond | Mother | 1-6 months postpartum | Maternal report | Mesosystem | Social support | Trimester 2 | Maternal report |
| Dubber et al. (2015) | Europe & Central Asia, N=30 | Caregiver felt bond | Mother | 1-6 months postpartum | Maternal report | Microsystem | Caregiver felt bond | Trimester 3 | Maternal report |
| Edhborg et al. (2011) | South Asia, N=672 | Caregiver felt bond | Mother | 1-6 months postpartum | Maternal report | Microsystem | Caregiver felt bond | Trimester 3 | Maternal report |
| Ensink et al. (2016) | North America, N=88 | Child attachment | Mother | 12-24 months postpartum | Researcher observation | Microsystem | Responsive caregiving | 6-12 months postpartum | Researcher observation |
| Favez et al. (2006) | Europe & Central Asia, N=39 | Caregiver-child interaction | Both/mixed | 1-6 months postpartum, 12-24 months postpartum | Researcher observation | Microsystem | Caregiver-child interaction, co-caregiving | Trimester 2, 1-6 months postpartum | Researcher observation |
| Feeley et al. (2005) | North America, N=72 | Responsive caregiving | Mother | 6-12 months postpartum | Researcher observation | Mesosystem | Social support | 1-6 months postpartum | Maternal report |
| Fernandes et al. (2021) | Europe & Central Asia, N=125 | Caregiver felt bond | Mother | 6-12 months postpartum | Maternal report | Microsystem | Caregiver felt bond | 1-6 months postpartum | Maternal report |
| Fijałkowska & Bielawska-Batorowicz (2020) | Europe & Central Asia, N=35 | Caregiver felt bond | Mother | 1-6 months postpartum | Maternal report, paternal report | Microsystem | Caregiver felt bond | Trimester 3 | Maternal report, paternal report |
| Fink et al. (2024) | Europe & Central Asia, N=93 | Caregiver-child interaction, responsive caregiving | Both/mixed, father, mother | 12-24 months postpartum, 6-12 months postpartum | Researcher observation | Microsystem | Responsive caregiving | 1-6 months postpartum | Researcher observation |
| Fish et al. (1993) | North America, N=76 | Responsive caregiving | Mother | 1-6 months postpartum | Researcher observation | Microsystem | Caregiver-partner relationship | <1 month postpartum | Maternal report |
| Florsheim & Smith (2005) | North America, N=36 | Responsive caregiving | Mother | 24-48 months postpartum | Researcher observation | Microsystem | Caregiver-partner relationship | Trimester 2 | Maternal report, paternal report |
| Forti-Buratti et al. (2017) | Europe & Central Asia, N=116 | Caregiver felt bond | Mother | 1-6 months postpartum | Maternal report | Microsystem | Caregiver felt bond | <1 month postpartum | Maternal report |
| Frosch et al. (2000) | North America, N=53 | Child attachment | Father, mother | 36-48 months postpartum | Maternal report, paternal report | Microsystem | Caregiver-partner relationship | 6-12 months postpartum | Researcher observation |
| Fuertes et al. (2006) | Europe & Central Asia, N=48 | Child attachment | Mother | 12-24 months postpartum | Researcher observation | Microsystem | Responsive caregiving | 6-12 months postpartum | Researcher observation |
| Fuertes et al. (2016) | Europe & Central Asia, N=82 | Child attachment | Father, mother | 12-24 months postpartum | Researcher observation | Microsystem | Responsive caregiving | 12-24 months postpartum, 6-12 months postpartum | Researcher observation |
| Fuertes et al. (2024) | Europe & Central Asia, N=213 | Child attachment | Mother | 12-24 months postpartum | Researcher observation | Microsystem | Caregiver-child interaction, responsive caregiving | 1-6 months postpartum, 6-12 months postpartum | Researcher observation |
| Galdiolo & Roskam (2016) | Europe & Central Asia, N=62 | Caregiver-child interaction | Both/mixed | 12-24 months postpartum | Researcher observation | Microsystem | Co-caregiving | 12-24 months postpartum | Parent report (unspecified/mixed) |
| Gerlach et al. (2022) | Europe & Central Asia, N=182 | Child attachment, responsive caregiving | Mother | 12-24 months postpartum | Researcher observation | Microsystem | Child attachment, responsive caregiving | 6-12 months postpartum | Researcher observation |
| Gloger-Tippelt & Huerkamp (1998) | Europe & Central Asia, N=28 | Child attachment | Mother | 12-24 months postpartum | Researcher observation | Microsystem | Caregiver-partner relationship | Trimester 2, 1-6 months postpartum | Maternal report, paternal report |
| Göbel et al. (2024) | Europe & Central Asia, N=509 | Caregiver felt bond | Mother | 1-6 months postpartum, 12-24 months postpartum, 6-12 months postpartum | Maternal report | Microsystem | Caregiver felt bond | Trimester 2, trimester 3 | Maternal report |
| Grossmann et al. (2002) | North America, N=49 | Child attachment, responsive caregiving | Father, mother | 24-36 months postpartum | Researcher observation | Microsystem | Child attachment, responsive caregiving | 12-24 months postpartum, 6-12 months postpartum | Researcher observation |
| Hadian et al. (2021) | Middle East & North Africa, N=316 | Child attachment | Mother | Trimester 3 | Maternal report | Microsystem | Caregiver-partner relationship | Trimester 2 | Maternal report |
| Hall et al. (2014) | North America, N=189 | Responsive caregiving | Father | 24-36 months postpartum | Researcher observation | Microsystem | Caregiver felt bond | 6-12 months postpartum | Researcher observation |
| Handelzalts et al. (2021) | Middle East & North Africa, N=293 | Caregiver felt bond | Mother | 1-6 months postpartum | Maternal report | Exosystem, microsystem | Caregiver felt bond, rooming in | <1 month postpartum | Maternal report, other |
| Hawkins et al. (2015) | North America, N=77 | Child attachment | Mother | 12-24 months postpartum | Researcher observation | Microsystem | Responsive caregiving | 6-12 months postpartum | Researcher observation |
| Howes & Hamilton (1992) | North America, N=47 | Child attachment | Mother, teacher | 24-36 months postpartum, 24-48 months postpartum | Researcher observation | Microsystem | Child attachment | 24-36 months postpartum, 24-48 months postpartum | Researcher observation |
| Hsiao et al. (2015) | North America, N=50 | Caregiver-child interaction | Mother | 24-48 months postpartum | Researcher observation | Microsystem | Child attachment | 1-6 months postpartum | Researcher observation |
| Hsu & Lavelli (2005) | North America, N=32 | Caregiver-child interaction | Mother | 1-6 months postpartum | Researcher observation | Macrosystem | Cultural differences | Antenatal (unspecified) | Other |
| Huth-Bocks et al. (2004) | North America, N=204 | Caregiver felt bond | Mother | 12-24 months postpartum | Researcher observation | Mesosystem, microsystem | Intimate partner violence, responsive caregiving, social support | Trimester 3 | Maternal report, researcher observation |
| Kim & Goodman (2024) | North America, N=234 | Child attachment, responsive caregiving | Mother | 12-24 months postpartum, 6-12 months postpartum | Researcher observation | Microsystem | Co-caregiving, responsive caregiving | 1-6 months postpartum, 6-12 months postpartum | Maternal report, researcher observation |
| Knappe et al. (2021) | Europe & Central Asia, N=76 | Caregiver felt bond | Father | 1-6 months postpartum | Paternal report | Microsystem | Caregiver-partner relationship | Trimester 2 | Paternal report |
| Kochanska et al. (2024) | North America, N=100 | Caregiver-child interaction | Father, mother | 24-48 months postpartum | Researcher observation | Microsystem | Responsive caregiving | 6-12 months postpartum | Researcher observation |
| Kornfield et al. (2021) | North America, N=833 | Caregiver felt bond | Mother | 1-6 months postpartum | Maternal report | Exosystem, mesosystem | Perceived neighborhood safety, social stress, social support | Trimester 2 | Maternal report |
| Kuo et al. (2013) | East Asia & Pacific, N=160 | Caregiver felt bond | Mother | Trimester 2 | Maternal report | Microsystem | Caregiver felt bond | Trimester 1 | Maternal report |
| Lamm et al. (2015) | Sub-Saharan Africa, N=178 | Responsive caregiving | Mother | 1-6 months postpartum | Researcher observation | Macrosystem | Cultural differences | 1-6 months postpartum | Other |
| Liang et al. (2015) | East Asia & Pacific, N=83 | Responsive caregiving | Mother | 12-24 months postpartum, 6-12 months postpartum | Researcher observation | Microsystem | Responsive caregiving | 6-12 months postpartum | Researcher observation |
| Liang et al. (2021) | East Asia & Pacific, N=60 | Child attachment, responsive caregiving | Mother | 12-24 months postpartum | Researcher observation | Microsystem | Co-caregiving | 6-12 months postpartum | Researcher observation |
| Lickenbrock & Braungart-Rieker (2015) | North America, N=117 | Child attachment | Father, mother | 12-24 months postpartum | Researcher observation | Microsystem | Caregiver-partner relationship, responsive caregiving | 1-6 months postpartum | Maternal report, paternal report, researcher observation |
| Lindstedt et al. (2021) | North America, N=99 | Caregiver-child interaction, responsive caregiving | Father | 1-6 months postpartum, 12-24 months postpartum | Researcher observation | Microsystem | Caregiver-partner relationship, responsive caregiving | Trimester 3, 1-6 months postpartum | Maternal report, paternal report, researcher observation |
| Lohaus et al. (2001) | Europe & Central Asia, N=20 | Responsive caregiving | Mother | 1-6 months postpartum, <1 month postpartum | Researcher observation | Microsystem | Responsive caregiving | 1-6 months postpartum, <1 month postpartum | Researcher observation |
| Maas et al. (2016) | Europe & Central Asia, N=273 | Caregiver felt bond, responsive caregiving | Mother | 6-12 months postpartum | Maternal report, researcher observation | Microsystem | Caregiver felt bond | Trimester 2 | Maternal report |
| Macturk et al. (1993) | North America, N=20 | Caregiver-child interaction | Mother | 12-24 months postpartum | Researcher observation | Mesosystem | Social support | 12-24 months postpartum | Maternal report |
| Madigan et al. (2015) | North America, N=84 | Caregiver felt bond, child attachment | Mother | 6-12 months postpartum | Researcher observation | Microsystem | Caregiver felt bond | Trimesters 2 and 3 | Researcher observation |
| Michałek-Kwiecień et al. (2022) | Europe & Central Asia, N=213 | Caregiver felt bond | Father, mother |  | Maternal report, paternal report | Microsystem | Caregiver felt bond, caregiver-partner relationship | Trimester 3 | Maternal report |
| Mills-Koonce et al. (2008) | North America, N=1140 | Responsive caregiving | Mother | 24-48 months postpartum | Researcher observation | Microsystem | Responsive caregiving | 12-24 months postpartum, 24-48 months postpartum, 6-12 months postpartum | Researcher observation |
| Mitsven et al. (2022) | North America, N=625 | Child attachment | Mother | 12-24 months postpartum | Researcher observation | Microsystem | Responsive caregiving | 1-6 months postpartum | Researcher observation |
| Mogan (1987) | North America, N=78 | Caregiver-child interaction | Mother | 6-12 months postpartum, <1 month postpartum | Researcher observation | Microsystem | Caregiver-child interaction | 1-6 months postpartum, <1 month postpartum | Researcher observation |
| Müller (1996) | North America, N=196 | Child attachment | Mother | 1-6 months postpartum | Maternal report | Microsystem | Caregiver felt bond | Antenatal (unspecified) | Maternal report |
| Murakami et al. (2022) | East Asia & Pacific, N=17999 | Caregiver felt bond | Mother | 1-6 months postpartum | Maternal report | Mesosystem | Social stress | Trimester 2 | Maternal report |
| Nasreen et al. (2022) | East Asia & Pacific, N=566 | Caregiver felt bond | Mother | 1-6 months postpartum | Maternal report | Microsystem | Intimate partner violence | Trimester 3 | Maternal report |
| Nuttall et al. (2015) | North America, N=374 | Responsive caregiving | Mother | 12-24 months postpartum, 6-12 months postpartum | Researcher observation | Microsystem | Responsive caregiving | 1-6 months postpartum, 6-12 months postpartum | Researcher observation |
| Ohara et al. (2018) | East Asia & Pacific, N=855 | Caregiver felt bond | Mother | 1-6 months postpartum | Maternal report | Mesosystem | Social support | Trimester 2 | Maternal report |
| Olsavsky et al. (2020) | North America, N=59 | Child attachment | Father | 12-24 months postpartum | Researcher observation | Microsystem | Responsive caregiving | 6-12 months postpartum | Researcher observation |
| Parfitt et al. (2014) | Europe & Central Asia, N=66 | Caregiver felt bond | Father, mother | 1-6 months postpartum, 12-24 months postpartum | Maternal report, paternal report | Microsystem | Caregiver felt bond, caregiver-partner relationship | Trimester 3, 1-6 months postpartum | Maternal report, paternal report |
| Pazzagli et al. (2022) | Europe & Central Asia, N=110 | Caregiver felt bond | Mother | 1-6 months postpartum | Maternal report | Microsystem | Caregiver felt bond | Trimester 3 | Maternal report |
| Planalp et al. (2013) | North America, N=130 | Responsive caregiving | Father, mother | 1-6 months postpartum, 6-12 months postpartum | Researcher observation | Microsystem | Responsive caregiving | 1-6 months postpartum | Researcher observation |
| Popp et al. (2008) | North America, N=212 | Responsive caregiving | Mother | 24-48 months postpartum | Researcher observation | Microsystem | Responsive caregiving | 12-24 months postpartum | Researcher observation |
| Raby et al. (2012) | North America, N=154 | Child attachment | Mother | 12-24 months postpartum | Researcher observation | Microsystem | Responsive caregiving | 6-12 months postpartum | Researcher observation |
| Riesch (1984) | North America, N=50 | Caregiver-child interaction, responsive caregiving | Mother | 6-12 months postpartum, <1 month postpartum | Researcher observation | Exosystem | Job satisfaction | Trimester 3 | Maternal report |
| Rossen et al. (2017) | East Asia & Pacific, N=372 | Caregiver felt bond | Mother | 1-6 months postpartum | Maternal report | Microsystem | Caregiver felt bond | Trimester 1, trimester 2, trimester 3 | Maternal report |
| Rossen et al. (2018) | East Asia & Pacific, N=382 | Caregiver-child interaction | Father, mother | 12-24 months postpartum | Researcher observation | Microsystem | Caregiver felt bond | 1-6 months postpartum | Maternal report, paternal report |
| Rubertsson et al. (2015) | Europe & Central Asia, N=718 | Caregiver felt bond | Mother | Trimester 3 | Maternal report | Mesosystem, microsystem | Caregiver-partner relationship, social support | Trimester 1 | Maternal report |
| Salo et al. (2021) | Europe & Central Asia, N=556 | Caregiver-child interaction | Both/mixed | 12-24 months postpartum | Maternal report, paternal report | Microsystem | Caregiver-partner relationship | 1-6 months postpartum | Maternal report, paternal report |
| Saravanan et al. (2023) | South Asia, N=91 | Caregiver felt bond | Mother | 1-6 months postpartum, <1 month postpartum | Maternal report | Microsystem | Caregiver felt bond, caregiver-partner relationship | Trimester 3 | Maternal report |
| Schaber et al. (2021) | Europe & Central Asia, N=637 | Caregiver felt bond | Father | 12-24 months postpartum | Paternal report | Microsystem | Caregiver-partner relationship | 1-6 months postpartum | Paternal report |
| Seifer et al. (1996) | North America, N=49 | Child attachment | Mother | 12-24 months postpartum | Researcher observation | Microsystem | Responsive caregiving | 6-12 months postpartum | Maternal report |
| Shannon et al. (2006) | North America, N=74 | Responsive caregiving | Father | 12-24 months postpartum | Researcher observation | Microsystem | Caregiver-partner relationship | 6-12 months postpartum | Paternal report |
| Shaw & Vondra (1995) | North America, N=100 | Child attachment | Mother | 12-24 months postpartum | Researcher observation | Microsystem | Responsive caregiving | 12-24 months postpartum | Researcher observation |
| Shawcroft et al. (2024) | North America, N=224 | Caregiver-child interaction, responsive caregiving | Both/mixed | 24-48 months postpartum | Researcher observation | Microsystem | Child attachment | 12-24 months postpartum | Maternal report |
| Sprangler et al. (1996) | North America, N=88 | Child attachment | Mother | 12-24 months postpartum | Researcher observation | Microsystem | Responsive caregiving | 6-12 months postpartum | Researcher observation |
| Stuijfzand et al. (2020) | Europe & Central Asia, N=488 | Caregiver felt bond | Father, mother | 1-6 months postpartum | Maternal report, paternal report | Mesosystem | Social support | Trimester 3 | Maternal report, paternal report |
| Takahashi (1990) | East Asia & Pacific, N=60 | Child attachment | Mother | 12-24 months postpartum | Researcher observation | Macrosystem | Cultural differences | Postnatal (unspecified) | Other |
| Tandberg et al. (2019) | Europe & Central Asia, N=132 | Caregiver felt bond | Father, mother | 1-6 months postpartum, <1 month postpartum | Maternal report, paternal report | Exosystem | Rooming in | <1 month postpartum | Other |
| Tani et al. (2018) | Europe & Central Asia, N=201 | Responsive caregiving | Mother | <1 month postpartum | Researcher observation | Microsystem | Caregiver felt bond | Trimester 3 | Maternal report |
| Taverna et al. (2024) | Latin America & Caribbean, N=32 | Caregiver-child interaction, responsive caregiving | Mother | 24-48 months postpartum | Researcher observation | Macrosystem | Cultural differences | 12-24 months postpartum | Other |
| Tian et al. (2023) | North America, N=125 | Caregiver-child interaction | Both/mixed | 24-48 months postpartum | Researcher observation | Microsystem | Caregiver-partner relationship, child attachment | 12-24 months postpartum | Researcher observation |
| Tichelman et al. (2020) | Europe & Central Asia, N=634 | Caregiver felt bond | Mother | 12-24 months postpartum | Maternal report | Mesosystem | Social support | Trimester 1 | Maternal report |
| Toivo et al. (2023) | Europe & Central Asia, N=100 | Caregiver felt bond | Father, mother | 6-12 months postpartum | Maternal report, paternal report | Microsystem | Caregiver felt bond | 1-6 months postpartum | Maternal report, paternal report |
| Udry-Jørgensen et al. (2011) | Europe & Central Asia, N=33 | Child attachment | Mother | 12-24 months postpartum | Researcher observation | Microsystem | Caregiver-child interaction, responsive caregiving | 1-6 months postpartum | Researcher observation |
| Van Bussel et al. (2010) | Europe & Central Asia, N=263 | Caregiver felt bond | Mother | 1-6 months postpartum | Maternal report | Microsystem | Caregiver felt bond | Trimester 3, trimesters 1 and 2 | Maternal report |
| Vernon-Feagans et al. (2016) | North America, N=1145 | Responsive caregiving | Mother | 12-24 months postpartum, 24-48 months postpartum, 6-12 months postpartum | Researcher observation | Microsystem | Responsive caregiving | 24-48 months postpartum | Researcher observation |
| Volling & Belsky (1992) | North America, N=113 | Child attachment | Father | 12-24 months postpartum | Researcher observation | Microsystem | Caregiver-partner relationship, responsive caregiving | Trimester 3, 1-6 months postpartum, 6-12 months postpartum | Paternal report, researcher observation |
| Von Klitzing, Simoni, Amsler, et al. (1999) | Europe & Central Asia, N=41 | Caregiver-child interaction | Father, mother | 1-6 months postpartum | Researcher observation | Microsystem | Caregiver-child interaction | Trimesters 2 and 3 | Other |
| Von Klitzing, Simoni, & Bürgin (1999) | North America, N=36 | Caregiver-child interaction | Father, mother | 1-6 months postpartum, 12-24 months postpartum | Researcher observation | Microsystem | Caregiver-partner relationship | Trimester 3 | Other |
| Vreeswijk et al. (2015) | Europe & Central Asia, N=308 | Caregiver felt bond | Father, mother | 6-12 months postpartum | Researcher observation | Microsystem | Caregiver felt bond | Trimester 2 | Researcher observation |
| Wan et al. (2017) | Europe & Central Asia, N=147 | Caregiver-child interaction, responsive caregiving | Mother | 12-24 months postpartum, 6-12 months postpartum | Researcher observation | Microsystem | Caregiver-child interaction, responsive caregiving | 1-6 months postpartum, 6-12 months postpartum | Researcher observation |
| Witte et al. (2020) | Middle East & North Africa, N=105 | Caregiver-child interaction, child attachment | Father, mother | 24-48 months postpartum, 6-12 months postpartum | Researcher observation | Microsystem | Caregiver-child interaction | Trimester 3, 6-12 months postpartum | Researcher observation |
| Wörmann et al. (2012) | Sub-Saharan Africa, N=44 | Caregiver-child interaction | Mother | 1-6 months postpartum | Researcher observation | Macrosystem | Cultural differences | Trimester 3 | Other |

**Reference list of included studies**

Abbasoglu, A., Varnali, H., Tekindal, M. A., & Pala, H. G. (2022). Being a mother under the COVID-19 pandemic lockdown: Evaluation of perinatal anxiety, prenatal attachment, and maternal-infant attachment. *American Journal of Perinatology*. https://doi.org/10.1055/a-1884-1260

Ahlfs-Dunn, S. M., Benoit, D., & Huth-Bocks, A. C. (2022). Intergenerational transmission of trauma from mother to infant: the mediating role of disrupted prenatal maternal representations of the child. *Attachment and Human Development*, *24*(2), 229–251. https://doi.org/10.1080/14616734.2021.1933769

Altenburger, L. E., Schoppe-Sullivan, S. J., & Kamp Dush, C. M. (2018). Associations Between Maternal Gatekeeping and Fathers’ Parenting Quality. *Journal of Child and Family Studies*, *27*(8), 2678–2689. https://doi.org/10.1007/s10826-018-1107-3

Baldisserotto, M. L., & Filha, M. M. T. (2023). Construct validity and reliability of the Brazilian version of the Maternal-Fetal Attachment Scale (MFAS): a proposal for a 12-item short version. *Cadernos de Saude Publica*, *39*(5). https://doi.org/10.1590/0102-311XEN133922

Bang, K. S., Lee, I., Kim, S., Yi, Y., Huh, I., Jang, S. Y., Kim, D., & Lee, S. (2020). Relation between mothers taekyo, prenatal and postpartum depression, and infants temperament and colic: A longitudinal prospective approach. *International Journal of Environmental Research and Public Health*, *17*(20), 1–13. https://doi.org/10.3390/ijerph17207691

Barnett, D., Clements, M., Kaplan-Estrin, M., McCaskill, J. W., Hunt, K. H., Butler, C. M., Schram, J. L., & Janisse, H. C. (2006). Maternal resolution of child diagnosis: Stability and relations with child attachment across the toddler to preschooler transition. *Journal of Family Psychology*, *20*(1), 100–107. https://doi.org/10.1037/0893-3200.20.1.100

Belsky, J., & Fearon, R. M. P. (2002). Early attachment security, subsequent maternal sensitivity, and later child development: Does continuity in development depend upon continuity of caregiving? *Human Development*, *4*(3), 361–387. https://doi.org/10.1080/14616730210167267

Belsky, J., Gilstrap, B., & Rovine, M. (1984). The Pennsylvania Infant and Family Development Project, I: Stability and Change in Mother-Infant and Father-Infant Interaction in a Family Setting at One, Three, and Nine Months. *Child Development*, *55*(3), 692–705. https://doi.org/10.2307/1130122

Bendel-Stenzel, L. C., An, D., & Kochanska, G. (2023). Revisiting the debate on effects of parental power-assertive control in two longitudinal studies: early attachment security as a moderator. *Attachment and Human Development*, *25*(5), 461–486. https://doi.org/10.1080/14616734.2023.2262979

Bernier, A., Jarry-Boileau, V., & Lacharité, C. (2014). Marital satisfaction and quality of father-child interactions: The moderating role of child gender. *Journal of Genetic Psychology*, *175*(2), 105–117. https://doi.org/10.1080/00221325.2013.799059

Bilgin, A., & Wolke, D. (2022). Bed-Sharing in the First 6 Months: Associations with Infant-Mother Attachment, Infant Attention, Maternal Bonding, and Sensitivity at 18 Months. *Journal of Developmental and Behavioral Pediatrics*, *43*(1), E9–E19. https://doi.org/10.1097/DBP.0000000000000966

Bloom, K. C. (1995). The development of attachment behaviors in pregnant adolescents. *Nursing Research*, *44*(5), 284–289. https://doi.org/10.1097/00006199-199509000-00005

Boekhorst, M. G. B. M., de Waal, N., Smit, L., Hulsbosch, L. P., van den Heuvel, M. I., Schwabe, I., Pop, V., & Nyklíček, I. (2024). A longitudinal study on the association between trait mindfulness and maternal bonding across the perinatal period. *Journal of Reproductive and Infant Psychology*. https://doi.org/10.1080/02646838.2024.2342904

Bornstein, M. H., Putnick, D. L., & Suwalsky, J. T. D. (2012). A Longitudinal Process Analysis of Mother-Child Emotional Relationships in a Rural Appalachian European American Community. *American Journal of Community Psychology*, *50*(1–2), 89–100. https://doi.org/10.1007/s10464-011-9479-1

Bornstein, M. H., Putnick, D. L., & Suwalsky, J. T. D. (2016). Emotional interactions in European American mother-infant firstborn and secondborn dyads: A within-family study. *Developmental Psychology*, *52*(9), 1363–1369. https://doi.org/10.1037/dev0000158

Bornstein, M. H., Putnick, D. L., & Suwalsky, J. T. D. (2019). Continuity, stability, and concordance of socioemotional functioning in mothers and their sibling children. *Social Development*, *28*(1), 90–105. https://doi.org/10.1111/sode.12319

Branjerdporn, G., Meredith, P., Wilson, T., & Strong, J. (2020). Prenatal Predictors of Maternal-infant Attachment. *Canadian Journal of Occupational Therapy*, *87*(4), 265–277. https://doi.org/10.1177/0008417420941781

Braungart-Rieker, J. M., Garwood, M. M., Powers, B. P., & Wang, X. (2001). Parental Sensitivity, Infant Affect, and Affect Regulation: Predictors of Later Attachment. *Child Development*, *72*(1), 252–270. https://research.ebsco.com/linkprocessor/plink?id=1e017f35-d83b-372f-944b-1898767f1dad

Britton, J. R., Britton, H. L., & Gronwaldt, V. (1999). Early perinatal hospital discharge and parenting during infancy. *Pediatrics*, *104*(5 I), 1070–1076. https://doi.org/10.1542/peds.104.5.1070

Capuzzi, C. (1989). Maternal attachment to handicapped infants and the relationship to social support. *Research in Nursing & Health*, *12*(3), 161–167. https://doi.org/10.1002/nur.4770120306

Cárcamo, R. A., Vermeer, H. J., van der Veer, R., & van IJzendoorn, M. H. (2016). Early Full-Time Day Care, Mother-Child Attachment, and Quality of the Home Environment in Chile: Preliminary Findings. *Early Education and Development*, *27*(4), 457–477. https://research.ebsco.com/linkprocessor/plink?id=b879d0b4-3175-3efb-9809-aa053efbe83f

Cataudella, S., Lampis, J., Busonera, A., Congia, F., Melis, G. B., & Zavattini, G. C. (2022). From pregnancy to 3 months after birth: The beginning of mother-infant relationship from a maternal perspective. *Journal of Reproductive and Infant Psychology*, *40*(3), 266–287. https://doi.org/10.1080/02646838.2021.1995597

Célia, M. G., Stack, D. M., & Serbin, L. A. (2018). Developmental patterns of change in mother and child emotional availability from infancy to the end of the preschool years: A four-wave longitudinal study. *Infant Behavior and Development*, *52*, 76–88. https://doi.org/10.1016/j.infbeh.2018.05.005

Chibucos, T. R., & Kail, P. R. (1981). Longitudinal Examination of Father-Infant Interaction and Infant-Father Attachment. *Merrill-Palmer Quarterly of Behavior and Development*, *27*(2), 81–96. https://research.ebsco.com/linkprocessor/plink?id=752af363-62d3-3456-a46d-5f9e9a57283b

Chtabou, G., Ahami, A., Boualam, A., Soufiani, A., Chkirate, M., Mammad, K., Rouane, J., Karjouh, K., Alami, M. H., Alaoui, A. M., & Azzaoui, F. Z. (2024). Post-partum depressive symptomatology and its possible effect on mother-baby bond in Morocco. *Activitas Nervosa Superior Rediviva*, *66*(3–4), 97–104. https://doi.org/10.31577/ansr.2024.66.3.1

Çınar, N., Yalnızoğlu Çaka, S., Topal, S., & Uslu Yuvacı, H. (2022). Relationship between prenatal and maternal attachment: a longitudinal study from Turkey. *Journal of Obstetrics and Gynaecology*, *42*(2), 220–227. https://doi.org/10.1080/01443615.2021.1904223

Condon, J., Corkindale, C., Boyce, P., & Gamble, E. (2013). A longitudinal study of father-to-infant attachment: antecedents and correlates. *Journal of Reproductive and Infant Psychology*, *31*(1), 15–30. https://doi.org/10.1080/02646838.2012.757694

Cox, M. J. (1992). Prediction of Infant-Father and Infant-Mother Attachment. *Developmental Psychology*, *28*(3), 474–483. https://research.ebsco.com/linkprocessor/plink?id=a5ae8053-b8b1-39ff-b6d2-a3239ded7409

Crockenberg, S. C., & Leerkes, E. M. (2003). Parental acceptance, postpartum depression, and maternal sensitivity: Mediating and moderating processes. *Journal of Family Psychology*, *17*(1), 80–93. https://doi.org/10.1037/0893-3200.17.1.80

Cuijlits, I., van de Wetering, A. P., Endendijk, J. J., van Baar, A. L., Potharst, E. S., & Pop, V. J. M. (2019). Risk and protective factors for pre- and postnatal bonding. *Infant Mental Health Journal*, *40*(6), 768–785. https://doi.org/10.1002/imhj.21811

da Rosa, K. M., Scholl, C. C., Ferreira, L. A., Trettim, J. P., da Cunha, G. K., Rubin, B. B., Martins, R. da L., Motta, J. V. dos S., Fogaça, T. B., Ghisleni, G., Pinheiro, K. A. T., Pinheiro, R. T., Quevedo, L. de A., & de Matos, M. B. (2021). Maternal-fetal attachment and perceived parental bonds of pregnant women. *Early Human Development*, *154*. https://doi.org/10.1016/j.earlhumdev.2021.105310

Damato, E. G. (2004). Prenatal attachment and other correlates of postnatal maternal attachment to twins. *Advances in Neonatal Care*, *4*(5), 274–291. https://doi.org/10.1016/j.adnc.2004.07.005

Dayton, C. J., Levendosky, A. A., Davidson, W. S., & Bogat, G. A. (2010). The child as held in the mind of the mother: The influence of prenatal maternal representations on parenting behaviors. *Infant Mental Health Journal*, *31*(2), 220–241. https://doi.org/10.1002/imhj.20253

Diniz, E., DeSousa, D., Koller, S. H., & Volling, B. L. (2016). Longitudinal effects of contextual and proximal factors on mother-infant interactions among Brazilian adolescent mothers. *Infant Behavior and Development*, *43*, 36–43. https://doi.org/10.1016/j.infbeh.2016.02.002

Doyle, F. L., Dickson, S. J., Eapen, V., Frick, P. J., Kimonis, E. R., Hawes, D. J., Moul, C., Richmond, J. L., Mehta, D., & Dadds, M. R. (2023). Towards Preventative Psychiatry: Concurrent and Longitudinal Predictors of Postnatal Maternal-Infant Bonding. *Child Psychiatry and Human Development*, *54*(6), 1723–1736. https://doi.org/10.1007/s10578-022-01365-0

Dubber, S., Reck, C., Müller, M., & Gawlik, S. (2015). Postpartum bonding: the role of perinatal depression, anxiety and maternal–fetal bonding during pregnancy. *Archives of Women’s Mental Health*, *18*(2), 187–195. https://doi.org/10.1007/s00737-014-0445-4

Edhborg, M., Nasreen, H. E., & Kabir, Z. N. (2011). Impact of postpartum depressive and anxiety symptoms on mothers’ emotional tie to their infants 2-3 months postpartum: A population-based study from rural Bangladesh. *Archives of Women’s Mental Health*, *14*(4), 307–316. https://doi.org/10.1007/s00737-011-0221-7

Ensink, K., Normandin, L., Plamondon, A., Berthelot, N., & Fonagy, P. (2016). Intergenerational pathways from reflective functioning to infant attachment through parenting. *Canadian Journal of Behavioural Science / Revue Canadienne Des Sciences Du Comportement*, *48*(1), 9–18. https://doi.org/10.1037/cbs0000030

Favez, N., Frascarolo, F., Carneiro, C., Montfort, V., Corboz-Warnery, A., & Fivaz-Depeursinge, E. (2006). The development of the family alliance from pregnancy to toddlerhood and children outcomes at 18 months. *Infant and Child Development*, *15*(1), 59–73. https://doi.org/10.1002/icd.430

Feeley, N., Gottlieb, L., & Zelkowitz, P. (2005). Infant, Mother, and Contextual Predictors of Mother-Very Low Birth Weight Infant Interaction at 9 Months of Age. *Journal of Developmental and Behavioral Pediatrics*, *26*(1), 24–33. https://research.ebsco.com/linkprocessor/plink?id=6a044dc5-12b9-37e9-a719-85077c60c20e

Fernandes, D. V., Canavarro, M. C., & Moreira, H. (2021). The role of mothers’ self-compassion on mother–infant bonding during the COVID-19 pandemic: A longitudinal study exploring the mediating role of mindful parenting and parenting stress in the postpartum period. *Infant Mental Health Journal*, *42*(5), 621–635. https://doi.org/10.1002/imhj.21942

Fijałkowska, D., & Bielawska-Batorowicz, E. (2020). A longitudinal study of parental attachment: pre- and postnatal study with couples. *Journal of Reproductive and Infant Psychology*, *38*(5), 509–522. https://doi.org/10.1080/02646838.2019.1665172

Fink, E., Foley, S., Browne, W., & Hughes, C. (2024). Parental sensitivity and family conversation: A naturalistic longitudinal study with both mothers and fathers across three time-points in early infancy. *Infant Mental Health Journal*, *45*(4), 357–368. https://doi.org/10.1002/imhj.22117

Fish, M., Stifter, C. A., & Belsky, J. (1993). Early patterns of mother-infant dyadic interaction: Infant, mother, and family demographic antecedents. *Infant Behavior and Development*, *16*(1), 1–18.

Florsheim, P., & Smith, A. (2005). Expectant adolescent couples’ relations and subsequent parenting behavior. *Infant Mental Health Journal*, *26*(6), 533–548. https://doi.org/10.1002/imhj.20076

Forti-Buratti, M. A., Palanca-Maresca, I., Fajardo-Simón, L., Olza-Fernández, I., Bravo-Ortiz, M. F., & Marín-Gabriel, M. Á. (2017). Differences in mother-to-infant bonding according to type of C-section: Elective versus unplanned. *Early Human Development*, *115*, 93–98. https://doi.org/10.1016/j.earlhumdev.2017.09.020

Frosch, C. A., Mangelsdorf, S. C., & Mchale, J. L. (2000). Marital Behavior and the Security of Preschooler-Parent Attachment Relationships. *Journal of Family Psychology*, *14*(1), 144–161. https://doi.org/10.1037/0893-32O0.14.U44

Fuertes, M., Dos Santos, P. L., Beeghly, M., & Tronick, E. (2006). More than maternal sensitivity shapes attachment: Infant coping and temperament. *Annals of the New York Academy of Sciences*, *1094*, 292–296. https://doi.org/10.1196/annals.1376.037

Fuertes, M., Faria, A., Beeghly, M., & Lopes-dos-Santos, P. (2016). The effects of parental sensitivity and involvement in caregiving on mother-infant and father-infant attachment in a portuguese sample. *Journal of Family Psychology*, *30*(1), 147–156. https://doi.org/10.1037/fam0000139

Fuertes, M., Martelo, I., Almeida, R., Gonçalves, J. L., & Barbosa, M. (2024). Attachment and mother-infant interactions in dyads with infants born full-term, moderate-to-late preterm, and very-to-extreme preterm. *Early Human Development*, *189*. https://doi.org/10.1016/j.earlhumdev.2024.105943

Galdiolo, S., & Roskam, I. (2016). From me to us: The construction of family alliance. *Infant Mental Health Journal*, *37*(1), 29–44. https://doi.org/10.1002/imhj.21543

Gerlach, J., Fößel, J. M., Vierhaus, M., Sann, A., Eickhorst, A., Zimmermann, P., & Spangler, G. (2022). Family risk and early attachment development: The differential role of parental sensitivity. *Infant Mental Health Journal*, *43*(2), 340–356. https://doi.org/10.1002/imhj.21964

Gloger-Tippelt, G. S., & Huerkamp, M. (1998). Relationship Change at the Transition to Parenthood and Security of Infant-Mother Attachment. *International Journal of Behavioral Development*, *22*(3), 633–655. https://doi.org/https://doi.org/10.1080/016502598384306

Göbel, A., Lüersen, L., Asselmann, E., Arck, P., Diemert, A., Garthus-Niegel, S., Mudra, S., & Martini, J. (2024). Psychometric properties of the Maternal Postnatal Attachment Scale and the Postpartum Bonding Questionnaire in three German samples. *BMC Pregnancy and Childbirth*, *24*(1). https://doi.org/10.1186/s12884-024-06964-4

Grossmann, K., Grossmann E., K., Fremmer-Bombik, E., Kindler, H., Scheuerer-English, H., & Zimmermann, P. (2002). The Uniqueness of the Child-Father Attachment Relationship: Fathers’ Sensitive and Challenging Play as a Pivotal Variable in a 16-Year Longitudinal Study. *Social Development*, *11*(3), 307–331. https://research.ebsco.com/linkprocessor/plink?id=f6727ef7-e279-30bf-81b2-0acfae625e7b

Hadian, T., Moosavi, S., Meedya, S., Mohammad-Alizadeh-Charandabi, S., Mohammadi, E., & Mirghafourvand, M. (2021). Relationship of health practices with depression and maternal-fetal attachment in adolescent pregnant women: A prospective study. *Archives of Psychiatric Nursing*, *35*(5), 465–471. https://doi.org/10.1016/j.apnu.2021.06.011

Hall, R. A. S., De Waard, I. E. M., Tooten, A., Hoffenkamp, H. N., Vingerhoets, A. J. J. M., & van Bakel, H. J. A. (2014). From the father’s point of view: How father’s representations of the infant impact on father-infant interaction and infant development. *Early Human Development*, *90*(12), 877–883. https://doi.org/10.1016/j.earlhumdev.2014.09.010

Handelzalts, J. E., Levy, S., Molmen-Lichter, M., Muzik, M., Krissi, H., Wiznitzer, A., & Peled, Y. (2021). Associations of rooming-in with maternal postpartum bonding: the impact of mothers’ pre-delivery intentions. *Midwifery*, *95*. https://doi.org/10.1016/j.midw.2021.102942

Hawkins, E., Madigan, S., Moran, G., & Pederson, D. R. (2015). Mediating and moderating processes underlying the association between maternal cognition and infant attachment. *Journal of Applied Developmental Psychology*, *39*, 24–33. https://doi.org/10.1016/j.appdev.2015.04.001

Howes, C., & Hamilton, C. E. (1992). Children’s Relationships with Child Care Teachers: Stability and Concordance with Parental Attachments. *Child Development*, *63*(4), 867–878. https://doi.org/https://doi.org/10.2307/1131239

Hsiao, C., Koren-Karie, N., Bailey, H., & Moran, G. (2015). It takes two to talk: Longitudinal associations among infant–mother attachment, maternal attachment representations, and mother–child emotion dialogues. *Attachment and Human Development*, *17*(1), 43–64. https://doi.org/10.1080/14616734.2014.981671

Hsu, H. C., & Lavelli, M. (2005). Perceived and observed parenting behavior in American and Italian first-time mothers across the first 3 months. *Infant Behavior and Development*, *28*(4), 503–518. https://doi.org/10.1016/j.infbeh.2005.09.001

Huth-Bocks, A. C., Levendosky, A. A., Bogat, G. A., & von Eye, A. (2004). The Impact of Maternal Characteristics and Contextual Variables on Infant-Mother Attachment. *Child Development*, *75*(2), 480–496. https://research.ebsco.com/linkprocessor/plink?id=ae9f3eaa-b936-3ac6-b198-7189ba0c9b66

Kim, C. Y., & Goodman, S. H. (2024). Satisfaction With Parental Responsibilities and Disorganized Attachment Among Infants of Mothers at Risk for Depression. *Journal of Family Psychology*, *38*(2), 212–222. https://doi.org/10.1037/fam0001179

Knappe, S., Petzoldt, J., Garthus-Niegel, S., Wittich, J., Puls, H. C., Huttarsch, I., & Martini, J. (2021). Associations of Partnership Quality and Father-to-Child Attachment During the Peripartum Period. A Prospective-Longitudinal Study in Expectant Fathers. *Frontiers in Psychiatry*, *12*. https://doi.org/10.3389/fpsyt.2021.572755

Kochanska, G., Bendel-Stenzel, L., An, D., & Sivagurunathan, N. (2024). Early relational origins of Theory of Mind: A two-study replication. *Journal of Child Psychology and Psychiatry and Allied Disciplines*. https://doi.org/10.1111/jcpp.14029

Kornfield, S. L., White, L. K., Waller, R., Njoroge, W., Barzilay, R., Chaiyachati, B. H., Himes, M. M., Rodriguez, Y., Riis, V., Simonette, K., Elovitz, M. A., & Gur, R. E. (2021). Risk and resilience factors influencing postpartum depression and mother-infant bonding during COVID-19. *Health Affairs*, *40*(10), 1566–1574. https://doi.org/10.1377/hlthaff.2021.00803

Kuo, P. C., Bowers, B., Chen, Y. C., Chen, C. H., Tzeng, Y. L., & Lee, M. S. (2013). Maternal-foetal attachment during early pregnancy in Taiwanese women pregnant by in vitro fertilization. *Journal of Advanced Nursing*, *69*(11), 2502–2513. https://doi.org/10.1111/jan.12135

Lamm, B., Gudi, H., Fassbender, I., Freitag, C., Graf, F., Goertz, C., Spangler, S., Teubert, M., Knopf, M., Lohaus, A., Schwarzer, G., & Keller, H. (2015). Rural Nso and German middle-class mothers’ interaction with their 3- and 6-month-old infants: A longitudinal cross-cultural analysis. *Journal of Family Psychology*, *29*(4), 649–655. https://doi.org/10.1037/fam0000100

Liang, X., Lin, Y., Van IJzendoorn, M. H., & Wang, Z. (2021). Grandmothers are part of the parenting network, too! A longitudinal study on coparenting, maternal sensitivity, child attachment and behavior problems in a Chinese sample. *New Directions for Child and Adolescent Development*, *2021*(180), 95–116. https://doi.org/10.1002/cad.20442

Liang, X., Wang, Z. Y., Liu, H. Y., Lin, Q., Wang, Z., & Liu, Y. (2015). Adult attachment status predicts the developmental trajectory of maternal sensitivity in new motherhood among Chinese mothers. *Midwifery*, *31*(1), 68–73. https://doi.org/10.1016/j.midw.2014.05.011

Lickenbrock, D. M., & Braungart-Rieker, J. M. (2015). Examining antecedents of infant attachment security with mothers and fathers: An ecological systems perspective. *Infant Behavior and Development*, *39*, 62–73. https://doi.org/10.1016/j.infbeh.2015.03.003

Lindstedt, J., Korja, R., Vilja, S., & Ahlqvist-Björkroth, S. (2021). Fathers’ prenatal attachment representations and the quality of father–child interaction in infancy and toddlerhood. *Journal of Family Psychology*, *35*(4), 478–488. https://doi.org/10.1037/fam0000813

Lohaus, A., Keller, H., & Voelker, S. (2001). Relationships between eye contact, maternal sensitivity, and infant crying. *International Journal of Behavioral Development*, *25*(6), 542–548. https://doi.org/10.1080/01650250042000528

Maas, A. J. B. M., de Cock, E. S. A., Vreeswijk, C. M. J. M., Vingerhoets, A. J. J. M., & van Bakel, H. J. A. (2016). A longitudinal study on the maternal–fetal relationship and postnatal maternal sensitivity. *Journal of Reproductive and Infant Psychology*, *34*(2), 110–121. https://doi.org/10.1080/02646838.2015.1112880

Macturk, R. H., Meadow-Orlans, K. P., Koester, L. S., & Spencer, P. E. (1993). Social Support, Motivation, Language, and Interaction: A Longitudinal Study of Mothers and Deaf Infants. *Source: American Annals of the Deaf*, *138*(1), 19–25.

Madigan, S., Hawkins, E., Plamondon, A., Moran, G., & Benoit, D. (2015). Maternal representations and infant attachment: An examination of the prototype hypothesis. *Infant Mental Health Journal*, *36*(5), 459–468. https://doi.org/10.1002/imhj.21527

Michałek-Kwiecień, J., Kaźmierczak, M., & Karasiewicz, K. (2022). Closeness with a partner and parental bond with a child during the transition to parenthood. *Midwifery*, *105*. https://doi.org/10.1016/j.midw.2021.103209

Mills-Koonce, W. R., Gariepy, J. L., Sutton, K., & Cox, M. J. (2008). Changes in maternal sensitivity across the first three years: Are mothers from different attachment dyads differentially influenced by depressive symptomatology? *Attachment and Human Development*, *10*(3), 299–317. https://doi.org/10.1080/14616730802113612

Mitsven, S. G., Prince, E. B., Messinger, D. S., Tenenbaum, E. J., Sheinkopf, S. J., Tronick, E. Z., Seifer, R., & Lester, B. M. (2022). Testing the mid-range model: Attachment in a high risk sample. *Developmental Science*, *25*(3). https://doi.org/10.1111/desc.13185

Mogan, J. (1987). What can nurses learn from structured observations of mother-infant interactions? *Issues in Comprehensive Pediatric Nursing*, *10*(1), 67–73. https://doi.org/10.3109/01460868709029849

Müller, M. E. (1996). Prenatal and postnatal attachment: a modest correlation. *Journal of Journal of Obstetric, Gynecologic, and Neonatal Nursing*, *25*(2), 161–166. https://doi.org/10.1111/j.1552-6909.1996.tb02420.x

Murakami, K., Ishikuro, M., Obara, T., Ueno, F., Noda, A., Onuma, T., Matsuzaki, F., Kikuchi, S., Kobayashi, N., Hamada, H., Iwama, N., Metoki, H., Kikuya, M., Saito, M., Sugawara, J., Tomita, H., Yaegashi, N., & Kuriyama, S. (2022). Social isolation and postnatal bonding disorder in Japan: The Tohoku Medical Megabank Project Birth and Three-Generation Cohort Study. *Archives of Women’s Mental Health*, *25*(6), 1079–1086. https://doi.org/10.1007/s00737-022-01266-0

Nasreen, H. E., Pasi, H. B., Aris, M. A. M., Rahman, J. A., Rus, R. M., & Edhborg, M. (2022). Impact of parental perinatal depressive and anxiety symptoms trajectories on early parent-infant impaired bonding: a cohort study in east and west coasts of Malaysia. *Archives of Women’s Mental Health*, *25*(2), 377–387. https://doi.org/10.1007/s00737-021-01165-w

Nuttall, A. K., Valentino, K., Wang, L., Lefever, J. B., & Borkowski, J. G. (2015). Maternal history of parentification and warm responsiveness: The mediating role of knowledge of infant development. *Journal of Family Psychology*, *29*(6), 863–872. https://doi.org/10.1037/fam0000112

Ohara, M., Nakatochi, M., Okada, T., Aleksic, B., Nakamura, Y., Shiino, T., Yamauchi, A., Kubota, C., Morikawa, M., Murase, S., Goto, S., Kanai, A., Kato, R., Ando, M., & Ozaki, N. (2018). Impact of perceived rearing and social support on bonding failure and depression among mothers: A longitudinal study of pregnant women. *Journal of Psychiatric Research*, *105*, 71–77. https://doi.org/10.1016/j.jpsychires.2018.09.001

Olsavsky, A. L., Berrigan, M. N., Schoppe-Sullivan, S. J., Brown, G. L., & Kamp Dush, C. M. (2020). Paternal stimulation and father-infant attachment. *Attachment and Human Development*, *22*(1), 15–26. https://doi.org/10.1080/14616734.2019.1589057

Parfitt, Y., Ayers, S., Pike, A., Jessop, D. C., & Ford, E. (2014). A prospective study of the parent–baby bond in men and women 15 months after birth. *Journal of Reproductive and Infant Psychology*, *32*(5), 441–456. https://doi.org/10.1080/02646838.2014.956301

Pazzagli, C., Buratta, L., Cenci, G., Coletti, E., Giuliani, M. L., & Mazzeschi, C. (2022). Does Parental Reflective Functioning Mediate the Associations between the Maternal Antenatal and Postnatal Bond with the Child in a Community Sample? *International Journal of Environmental Research and Public Health*, *19*(12). https://doi.org/10.3390/ijerph19126957

Planalp, E. M., Braungart-Rieker, J. M., Lickenbrock, D. M., & Zentall, S. R. (2013). Trajectories of parenting during infancy: The role of infant temperament and marital adjustment for mothers and fathers. *Infancy*, *18*, E16–E45. https://doi.org/10.1111/infa.12021

Popp, T. K., Spinrad, T. L., & Smith, C. L. (2008). The relation of cumulative demographic risk to mothers’ responsivity and control: Examining the role of toddler temperament. *Infancy*, *13*(5), 496–518. https://doi.org/10.1080/15250000802329446

Raby, K. L., Cicchetti, D., Carlson, E. A., Cutuli, J. J., Englund, M. M., & Egeland, B. (2012). Genetic and caregiving-based contributions to infant attachment: Unique associations with distress reactivity and attachment security. *Psychological Science*, *23*(9), 1016–1023. https://doi.org/10.1177/0956797612438265

Riesch, S. K. (1984). Occupational Commitment and the Quality of Maternal Infant Interaction. *Research in Nursing & Health*, *7*(4), 295–303. https://doi.org/10.1002/nur.4770070408

Rossen, L., Hutchinson, D., Wilson, J., Burns, L., Allsop, S., Elliott, E. J., Jacobs, S., MacDonald, J. A., Olsson, C., & Mattick, R. P. (2017). Maternal Bonding through Pregnancy and Postnatal: Findings from an Australian Longitudinal Study. *American Journal of Perinatology*, *34*(8), 808–817. https://doi.org/10.1055/s-0037-1599052

Rossen, L., Mattick, R. P., Wilson, J., Burns, L., Macdonald, J. A., Olsson, C., Allsop, S., Elliott, E. J., Jacobs, S., McCormack, C., & Hutchinson, D. (2018). Mother–Infant and Partner–Infant Emotional Availability at 12 Months of Age: Findings From an Australian Longitudinal Study. *Infancy*, *23*(6), 893–916. https://doi.org/10.1111/infa.12247

Rubertsson, C., Pallant, J. F., Sydsjö, G., Haines, H. M., & Hildingsson, I. (2015). Maternal depressive symptoms have a negative impact on prenatal attachment – findings from a Swedish community sample. *Journal of Reproductive and Infant Psychology*, *33*(2), 153–164. https://doi.org/10.1080/02646838.2014.992009

Salo, S. J., Pajulo, M., Vinzce, L., Raittila, S., Sourander, J., & Kalland, M. (2021). Parent Relationship Satisfaction and Reflective Functioning as Predictors of Emotional Availability and Infant Behavior. *Journal of Child and Family Studies*, *30*(5), 1214–1228. https://doi.org/10.1007/s10826-021-01934-2

Saravanan, V., Desai, G., & Satyanarayana, V. A. (2023). Antenatal predictors of postnatal maternal attachment and competence after assisted conception—a prospective cohort study in South India. *Archives of Women’s Mental Health*, *26*(4), 549–560. https://doi.org/10.1007/s00737-023-01340-1

Schaber, R., Kopp, M., Zähringer, A., Mack, J. T., Kress, V., & Garthus-Niegel, S. (2021). Paternal leave and father-infant bonding: Findings from the population-based cohort study DREAM. *Frontiers in Psychology*, *12*. https://doi.org/10.3389/fpsyg.2021.668028

Seifer, R., Schiller, M., Sameroff, A. J., Resnick, S., & Riordan, K. (1996). Attachment, Maternal Sensitivity, and Infant Temperament During the First Year of Life. *Developmental Psychology*, *32*(1), 12–25.

Shannon, J. D., Tamis-Lemonda, C. S., & Cabrera, N. J. (2006). Fathering in Infancy: Mutuality and Stability Between 8 and 16 Months. *Parenting: Science & Practice*, *6*(2/3), 167–188. https://doi.org/https://doi.org/10.1207/s15327922par0602&3_3

Shaw, D. S., & Vondra, J. I. (1995). Infant Attachment Security and Maternal Predictors of Early Behavior Problems: A Longitudinal Study of Low-Income Families. *Journal of Abnormal Child Psychology*, *23*(3), 335–357. https://doi.org/https://doi.org/10.1007/bf01447561

Shawcroft, J., Coyne, S. M., Linder, L., Clifford, B. N., & McDaniel, B. T. (2024). Attachment security and problematic media use in infancy: A longitudinal study in the United States. *Infancy*, *29*(2), 137–154. https://doi.org/10.1111/infa.12570

Sprangler, G., Fremmer-Bombik, E., & Grossmann, K. (1996). Social and Individual Determinants of Infant Attachment Security and Disorganization. *Infant Mental Health Journal*, *17*(2), 127–139. https://doi.org/10.1002/(SICI)1097-0355(199622)17:2&lt;127::AID-IMHJ3&gt;3.0.CO;2-N

Stuijfzand, S., Garthus-Niegel, S., & Horsch, A. (2020). Parental Birth-Related PTSD Symptoms and Bonding in the Early Postpartum Period: A Prospective Population-Based Cohort Study. *Frontiers in Psychiatry*, *11*. https://doi.org/10.3389/fpsyt.2020.570727

Takahashi, K. (1990). Are the Key Assumptions of the “Strange Situation” Procedure Universal? A View from Japanese Research. *Source: Human Development*, *33*(1), 23–30. https://about.jstor.org/terms

Tandberg, B. S., Flacking, R., Markestad, T., Grundt, H., & Moen, A. (2019). Parent psychological wellbeing in a single-family room versus an open bay neonatal intensive care unit. *PLoS ONE*, *14*(11). https://doi.org/10.1371/journal.pone.0224488

Tani, F., Castagna, V., & Ponti, L. (2018). Women who had positive relationships with their own mothers reported good attachments to their first child before and after birth. *Acta Paediatrica, International Journal of Paediatrics*, *107*(4), 633–637. https://doi.org/10.1111/apa.14162

Taverna, A., Padilla, M., & Waxman, S. (2024). How pervasive is joint attention? Mother-child dyads from a Wichi community reveal a different form of “togetherness.” *Developmental Science*, *27*(5). https://doi.org/10.1111/desc.13471

Tian, Z., Hazen, N., & Jacobvitz, D. B. (2023). Triadic family interactions at 2 years: The role of prenatal marital quality and infants’ attachment configuration with mother and father. *Attachment and Human Development*, *25*(5), 524–543. https://doi.org/10.1080/14616734.2023.2257677

Tichelman, E., Henrichs, J., Schellevis, F. G., Berger, M. Y., & Burger, H. (2020). Development of a risk classification model in early pregnancy to screen for suboptimal postnatal mother-to-infant bonding: A prospective cohort study. *PLoS ONE*, *15*(11 November), 1–17. https://doi.org/10.1371/journal.pone.0241574

Toivo, J., Tulivuo, N., Kanzaki, M., Koivisto, A. M., Kylmä, J., & Paavilainen, E. (2023). First-Time Parents’ Bonding with Their Baby: A Longitudinal Study on Finnish Parents during the First Eight Months of Parenthood. *Children*, *10*(11). https://doi.org/10.3390/children10111806

Udry-Jørgensen, L., Pierrehumbert, B., Borghini, A., Habersaat, S., Forcada-Guex, M., Ansermet, F., & Muller-Nix, C. (2011). Quality of attachment, perinatal risk, and mother-infant interaction in a high-risk premature sample. *Infant Mental Health Journal*, *32*(3), 305–318. https://doi.org/10.1002/imhj.20298

Van Bussel, J. C. H., Spitz, B., & Demyttenaere, K. (2010). Three self-report questionnaires of the early mother-to-infant bond: Reliability and validity of the Dutch version of the MPAS, PBQ and MIBS. *Archives of Women’s Mental Health*, *13*(5), 373–384. https://doi.org/10.1007/s00737-009-0140-z

Vernon-Feagans, L., Willoughby, M., & Garrett-Peters, P. (2016). Predictors of behavioral regulation in kindergarten: Household chaos, parenting, and early executive functions. *Developmental Psychology*, *52*(3), 430–441. https://doi.org/10.1037/dev0000087

Volling, B. L., & Belsky, J. (1992). Infant, Father, and Marital Antecedents of Infant-Father Attachment Security in Dual-Earner and Single-Earner Families. *International Journal of Behavioral Development*, *15*(1), 83–100. https://research.ebsco.com/linkprocessor/plink?id=c72c3fe0-9119-324f-ae71-7ac3e9e8e5d4

Von Klitzing, K., Simoni, H., Amsler, F., & Bürgin, D. (1999). The role of the father in early family interactions. *Infant Mental Health Journal*, *20*(3), 222–237. https://doi.org/10.1002/(SICI)1097-0355(199923)20:3<222::AID-IMHJ2>3.0.CO;2-B

Von Klitzing, K., Simoni, H., & Bürgin, D. (1999). Child development and early triadic relationships. *International Journal of Psycho-Analysis*, *80*(1), 71–89. https://doi.org/10.1516/0020757991598576

Vreeswijk, C. M. J. M., Rijk, C. H. A. M., Maas, A. J. B. M., & van Bakel, H. J. A. (2015). Fathers’ and mothers’ representations of the infant: Associations with prenatal risk factors. *Infant Mental Health Journal*, *36*(6), 599–612. https://doi.org/10.1002/imhj.21541

Wan, M. W., Brooks, A., Green, J., Abel, K., & Elmadih, A. (2017). Psychometrics and validation of a brief rating measure of parent-infant interaction: Manchester assessment of caregiver-infant interaction. *International Journal of Behavioral Development*, *41*(4), 542–549. https://doi.org/10.1177/0165025416631835

Witte, A. M., Bakermans-Kranenburg, M. J., van IJzendoorn, M. H., Szepsenwol, O., & Shai, D. (2020). Predicting infant–father attachment: the role of pre- and postnatal triadic family alliance and paternal testosterone levels. *Attachment and Human Development*, *22*(6), 653–667. https://doi.org/10.1080/14616734.2019.1680713

Wörmann, V., Holodynski, M., Kärtner, J., & Keller, H. (2012). A cross-cultural comparison of the development of the social smile. A longitudinal study of maternal and infant imitation in 6- and 12-week-old infants. *Infant Behavior and Development*, *35*(3), 335–347. https://doi.org/10.1016/j.infbeh.2012.03.002
